# Biodegradable targeted polymeric mRNA nanoparticles enable in vivo CD19 CAR T cell generation and lead to B cell depletion

**DOI:** 10.1126/sciadv.adz1722

**Published:** 2026-03-11

**Authors:** Manav Jain, Savannah E. Est-Witte, Sydney R. Shannon, Sarah Y. Neshat, Xinjie Yu, Sydney Dunham, Tina Tian, Leonardo Cheng, Jawaun Harris, Maximilian F. Konig, Stephany Y. Tzeng, Jonathan P. Schneck, Jordan J. Green

**Affiliations:** ^1^ Department of Biomedical Engineering, Johns Hopkins University School of Medicine, Baltimore, MD 21231, USA.; ^2^ Institute for NanoBioTechnology, and Translational Tissue Engineering Center, Johns Hopkins University School of Medicine, Baltimore, MD 21231, USA.; ^3^ Johns Hopkins Translational ImmunoEngineering Center, Johns Hopkins University School of Medicine, Baltimore, MD 21231, USA.; ^4^ Department of Chemical and Biomolecular Engineering, Johns Hopkins University, Baltimore, MD 21218, USA.; ^5^ Division of Rheumatology, Department of Medicine, Johns Hopkins University School of Medicine, Baltimore, MD 21224, USA.; ^6^ Center for Autoimmunity and Immuno-Oncology, Johns Hopkins University School of Medicine, Baltimore, MD 21231, USA.; ^7^ Institute for Cell Engineering, Johns Hopkins University School of Medicine, Baltimore, MD 21231, USA.; ^8^ Departments of Pathology, Johns Hopkins University School of Medicine, Baltimore, MD 21231, USA.; ^9^ Department of Oncology, the Sidney Kimmel Comprehensive Cancer Center, and the Bloomberg~Kimmel Institute for Cancer Immunotherapy, Johns Hopkins University School of Medicine, Baltimore, MD 21231, USA.; ^10^ Department of Materials Science & Engineering, Johns Hopkins University, Baltimore, MD 21218, USA.; ^11^ Departments of Ophthalmology and Neurosurgery, Johns Hopkins University School of Medicine, Baltimore, MD 21231, USA.

## Abstract

While chimeric antigen receptor (CAR) T cell therapies have demonstrated therapeutic efficacy against B cell malignancies, widespread implementation of these therapies is hindered by a cumbersome, ex vivo manufacturing process. Delivery of CAR-encoding messenger RNA (mRNA) to endogenous T cells can generate these therapeutic cells in vivo and streamline this manufacturing workflow. To accomplish this, T cell–activating ligands were conjugated to a biodegradable polymeric mRNA nanoparticle to form T cell–targeted particles. By conjugating multiple activating ligands, T cell transfection and stimulation in vitro was increased, and greater T cell transfection and selectivity in vivo was achieved compared to an untargeted particle. These nanoparticles can flexibly encapsulate mRNA cargos and were used to deliver anti-CD19 CAR mRNA in vivo, enabling depletion of 95% of B cells in the peripheral blood and 50% depletion of splenic B cells in healthy mice. These findings regarding nanoparticle tropism and their potential therapeutic efficacy highlight the importance of this nonviral, polymeric platform to address key limitations associated with current CAR T practices.

## Teaser

Polymeric mRNA nanoparticles with stimulating ligands transfect T cells in vivo and elicit potent CAR-mediated B cell depletion.

## INTRODUCTION

Adoptive T cell immunotherapies have demonstrated remarkable clinical success due to their ability to perform potent, antigen-specific target cell killing (*1*). Of the different Food and Drug Administration (FDA)–approved T cell therapies, chimeric antigen receptor (CAR) T cell therapy is the most widely adopted, with six FDA-approved products as of early 2025 (*2*). These CAR T therapies are currently implemented in the clinic for the treatment of B cell lineage cancers through the targeting of CD19 or B cell maturation antigen. CAR T technology is now being investigated in the context of solid tumors, autoimmunity, and infectious disease across numerous clinical trials (*3*–*5*). Despite this proven clinical efficacy, widespread adoption of CAR T therapies is hindered by a cumbersome, time-intensive, and expensive ex vivo manufacturing process (*6*). For all approved CAR T therapies, autologous T cells must be isolated from patient blood, transported to a specialized engineering facility, genetically engineered using a viral vector to induce CAR expression, expanded ex vivo for ~14 days to reach a therapeutic dose, and then administered to the patient (*7*, *8*). In addition to this complex manufacturing process, patients must also undergo intensive conditioning therapy before CAR T infusion to facilitate engraftment and expansion (*9*). Furthermore, the integration of nontargeted viral vectors into the host genome has the potential to induce genomic instability and tumor suppressor gene loss that can lead to the development secondary T cell malignancies (*10*). Overall, the potential efficacy of CAR T therapies comes with notable logistical, health, and monetary concerns for the patient.

In vivo approaches to generating CAR T cells would address many of the above concerns and improve the accessibility of CAR T therapies (*11*). Reprogramming a patient’s endogenous T cell repertoire to express a CAR would avoid many of the roadblocks in the current manufacturing workflow, mainly the need for T cell isolation, ex vivo engineering, and expansion. While viral vectors for in vivo engineering are effective gene delivery tools, they are expensive to manufacture and highly immunogenic, meaning that they cannot be dosed repeatedly (*12*). On the other hand, the widespread implementation of lipid nanoparticle (LNP)–based mRNA COVID-19 vaccines highlights the potent ability of nonviral vectors to deliver mRNA in vivo across multiple doses (*13*, *14*). In addition to this proven efficacy, mRNA is a safe, appealing candidate for inducing CAR expression in vivo, as the transient nature of mRNA will avoid unwanted, permanent genomic integration into the patient’s endogenous T cells (*15*). Furthermore, transient CAR expression in vivo can avoid extended cytokine release and related toxicities that are often observed with permanently edited CAR T cells (*16*). This approach of using mRNA-engineered CAR T cells is already being investigated in the clinic with ex vivo CAR T therapy and adoptive transfer (*17*, *18*).

Despite potential of this reprogramming approach, T cells are historically a difficult cell type to transfect and engineer in vivo (*19*). Moreover, there are two key delivery challenges for effective transfection: selective targeting of and robust stimulation of T cells. Regarding the issue of selective targeting, systemically delivered NPs tend to accumulate in the liver and are generally taken up by cells in the mononuclear phagocytic system (*20*, *21*). Recent advancements in nonviral NP engineering have sought to improve delivery to hard-to-reach lymphoid organs and immune cell types by either modulating NP chemistry or by functionalizing the NP with a targeting ligand, such as an antibody (*22*–*26*). Regarding the need for T cell stimulation, ex vivo T cell activation is often accomplished with anti-CD3 (“signal 1”) and anti-CD28 (“signal 2”) antibody-coated beads (*27*–*29*). In addition to stimulating proliferation, this activation typically enables improved uptake of NP-based cargo. Upon uptake, mRNA NPs then need to undergo endosomal escape, release of mRNA cargo, and translation of the mRNA into a functional protein construct (*30*).

Previous literature has demonstrated that conjugating certain ligands to NPs can enable on-target gene delivery to T cells in vivo. Incorporation of antibodies such as anti-CD3 (*31*–*34*), anti-CD4 (*35*, *36*), anti-CD8 (*37*), anti-CD5 (*38*, *39*), and anti-CD7 (*40*) into the NP surface are all effective ways to transfect endogenous T cells with lipid and polymer-based nonviral vectors. While these targeted NPs have proven efficacy in transfecting T cells, their ability to serve as platforms for inducing T cell activation is less characterized. Together, there is a great need for an mRNA NP-based platform that can both stimulate and transfect T cells in vivo to generate functional CAR T cells.

**Fig. 1.**
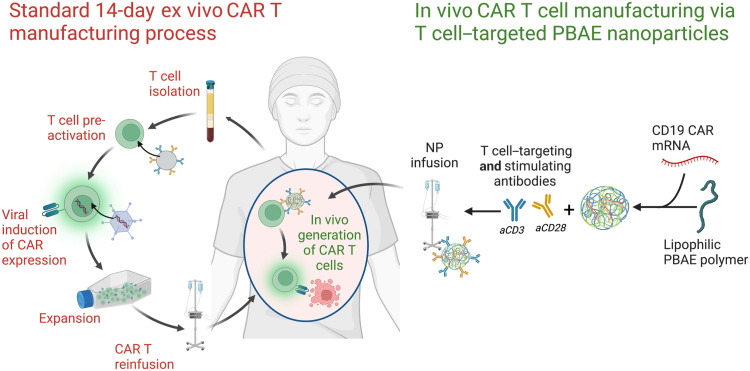
tPNPs can generate CAR T cells in a simpler, faster process compared to conventional ex vivo CAR T manufacturing. In standard, ex vivo CAR T cell therapy, T cells are isolated from patient blood and activated using biomaterial systems that present anti-CD3 and anti-CD28. Activated T cells are then transduced with a viral vector to permanently express a CAR. These cells are expanded for 10 to 14 days until a therapeutic dose of CAR T cells is reached before they are reinfused into the patient. In the proposed system of tPNP-mediated in vivo CAR T cell generation, NPs composed of a PBAE polymer, a functional lipid linker, and mRNA encoding a CAR construct are fabricated and combined with anti-CD3 and anti-CD28 antibodies. Upon infusion of tPNPs into a patient, these particles will target, activate, and transfect endogenous T cells to generate functional CAR T cells. In both ex vivo and in vivo systems, the final CAR T cell will then kill its target CD19^+^ B cell.

Here, we develop and characterize a targeted polymeric NP platform for in vivo T cell transfection and CAR T cell generation (Fig. 1). We used poly(beta-amino ester) (PBAE)–based NPs to deliver mRNA to immune cells in vitro and in vivo (*37*, *41*). PBAEs are a class of cationic polymers that can self-assemble with nucleic acids to form NPs; contain primary, secondary, and tertiary amines to facilitate intracellular delivery; and are highly modular, enabling tuning of polymer chemistry to optimize delivery to a specific cell type (*42*). PBAEs have recently shown the ability to boost endosomal escape by up to an order of magnitude compared to several commercially available polymer and lipid-based nanomaterials (*43*). Uptake and transfection into T cells with NPs has historically been a challenge in the field, and incorporation of charged polyelectrolyte ligands on the surface of charged polyelectrolyte complexes can be difficult to manufacture as performance is very sensitive to charge ratio (*22*, *44*, *45*). Thus, we fabricated a nanostructured construct designed for T cell engineering composed of (i) a next-generation lipophilic-hydrophilic PBAE copolymer optimized for efficient endosomal escape and quick hydrolytic degradation to overcome the downstream barriers of gene delivery and (ii) lipid-anchored polyethylene glycol (PEG)–conjugated ligands to improve the upstream barriers of T cell–specific NP uptake and promotion of selective T cell activation. Of note, rather than necessitating four to five separate biomaterial components to construct next-generation targeted LNPs, these polymeric NPs (PNPs) only require the PBAE polymer, a PEG lipid, and the mRNA cargo (*23*, *46*). We conjugated ligands anti-CD3 (aCD3) and anti-CD28 (aCD28) to the PBAE-mRNA NP surface to form T cell–targeted PNPs (tPNPs). By conjugating signal 1 and 2 ligands to the particle, we were able to achieve robust T cell transfection and activation in vitro. We additionally demonstrate that aCD3+aCD28-coated tPNPs are capable of robust T cell–specific transfection and stimulation in vivo. Furthermore, incorporation of dual targeting ligands alters the tropism of systemically delivered tPNPs toward lymphoid organs and T cells and away from the liver and macrophages. Last, we demonstrate that tPNP-mediated delivery of mRNA encoding a therapeutic anti-CD19 CAR construct can elicit robust B cell depletion in the peripheral blood and spleens of healthy mice. We characterize the temporal kinetics of this B cell depletion and effect of repeated dosing to demonstrate the therapeutic potential of this platform.

## RESULTS

### Formulation of tPNPs

**Fig. 2.**
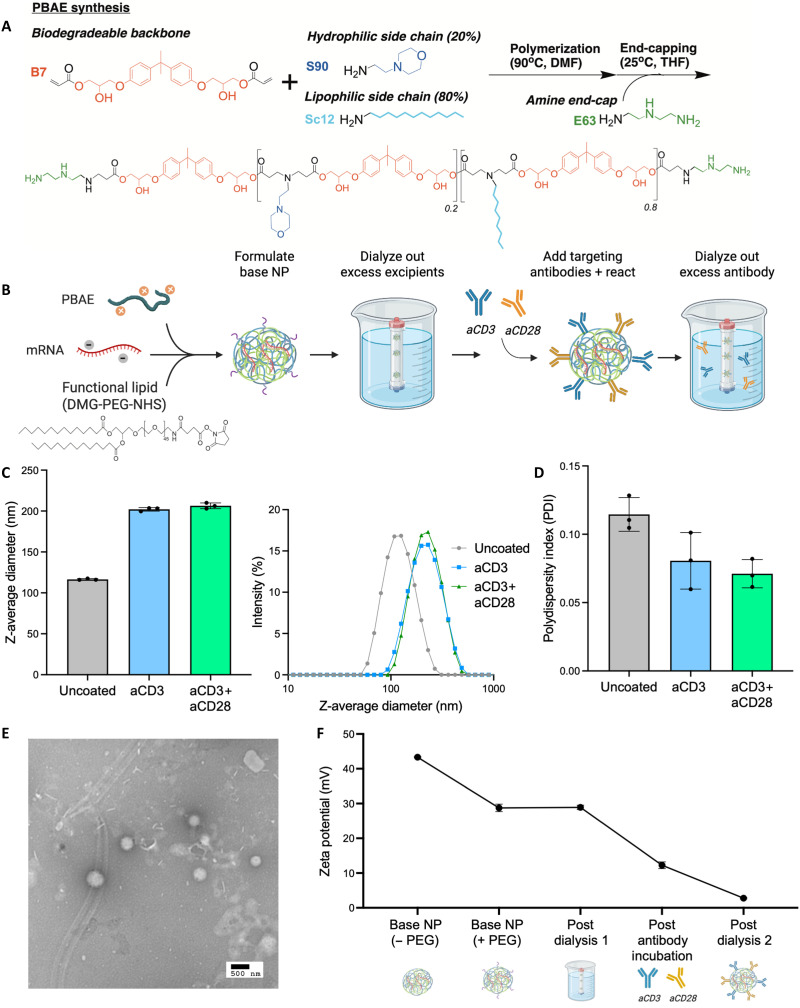
tPNP formulation and characterization. (**A**) Overview of PBAE synthesis. A base polymer was formed by reacting a biodegradable diacrylate backbone monomer (B7) with a 20:80 mixture of a hydrophilic amine side-chain monomer (S90) and a lipophilic amine side-chain monomer (Sc12) via Michael addition. The base polymer is then end-capped with an excess of amine-containing monomer (E63) to form the final PBAE. (**B**) Overview of targeted PBAE NP formulation. The lipophilic PBAE synthesized in (A) was then complexed with mRNA to form a base NP, and DMG-PEG-NHS was incorporated into the NP via postinsertion. After dialysis into PBS, the base NPs were reacted with a 1:1 mixture of T cell–targeting antibodies, anti-CD3, and anti-CD28. Excess antibody was removed from the NP via dialysis. (**C** and **D**) Characterization of tPNP hydrodynamic diameter via DLS. NPs were diluted in saline, and size was analyzed in triplicate, with representative size curves shown. (D) Polydispersity of tPNP hydrodynamic diameter was analyzed via DLS in triplicate. (**E**) Dry diameter of aCD3+aCD28 tPNPs was analyzed via TEM, with a representative image shown, Scale bar, 500 nm. (**F**) Characterization of tPNP surface charge. NPs were diluted in 0.1× saline and analyzed in triplicate during each phase of tPNP synthesis.

To formulate PBAE NPs for T cell transfection, we synthesized a hydrophilic-lipophilic PBAE blend via a two-step Michael addition whereby a diacrylate backbone monomer was reacted with hydrophilic and lipophilic amine sidechain monomers to form a base polymer (Fig. 2A) (*41*). The base polymer was then terminated with an amine-containing endcap monomer to form the final PBAE (*47*). This PBAE structure, bisphenol A glycerolate diacrylate (B7)–4-(2-Aminoethyl)morpholine (S90), 1-dodecylamine (Sc12) (80%)–diethylenetriamine (E63), was selected in part because 80% of the polymer’s sidechains were composed of the lipophilic monomer, 1-dodecylamine. This lipophilic nature allowed for incorporation of a functional lipid-anchored PEG molecule into the NP surface through hydrophobic interactions in a manner similar to PEG incorporation in LNPs (*48*, *49*). Base PBAE NPs were formulated by mixing the PBAE with mRNA and a functional PEG–*N-*hydroxysuccinimide (NHS) lipid that was incorporated via the postinsertion technique (Fig. 2B). After purifying the base NP, targeting ligands anti-CD3 (aCD3) and/or anti-CD28 (aCD28) were conjugated to the functional NHS groups displayed on the NP surface to form tPNPs. Following the reaction, any unreacted antibody was removed via dialysis.

tPNP hydrodynamic diameter was analyzed via dynamic light scattering (DLS), revealing that base particles that were not conjugated with antibody (“Uncoated”) were smaller (117 ± 1 nm) than particles coated with aCD3 or aCD3+aCD28 tPNPs (202 ± 1 and 206 ± 2 nm, respectively) (Fig. 2C). This growth in NP size after ligand incubation has been previously reported and is most likely due to the successful conjugation of antibody to the NP surface (*32*). All NP groups had an average polydispersity index below 0.12, indicating particle uniformity (Fig. 2D). Moreover, analysis of tPNP hydrodynamic radius and size distribution via NP tracking analysis further supported the finding that the uncoated and aCD3+aCD28 tPNP were similar in size, with the antibody-conjugated NP being ~10 nm larger in diameter (fig. S1). Analysis of dried tPNP size via transmission electron microscopy (TEM) further confirmed that the final size of the antibody-conjugated NP is approximately 200 nm (Fig. 2E).

tPNP zeta potential was also measured over the course of NP formulation (Fig. 2F). Although the base polymer-mRNA NP has a highly positive zeta potential (43.3 ± 0.1 mV), addition of the 1,2-dimyristoyl-rac-glycero-3–PEG-2000–NHS (DMG-PEG-NHS) and antibody cause the NP surface charge to decrease to 28.7 ± 0.6 and 12.3 ± 0.6 mV, respectively. Following the final NP dialysis/purification into phosphate-buffered saline (PBS), the NP surface charge is approximately neutral (2.8 ± 0.1 mV), which has previously been shown to be ideal for systemic delivery applications (*50*). Particle size was also characterized across a variety of storage conditions and times, revealing that tPNPs begin to increase in size and aggregate after 30 days of being stored at −80°C or undergoing 10 or more freeze-thaw cycles (fig. S2). Thus, all tPNPs were used within 14 days of formulation and within three freeze-thaw cycles. In addition, tPNPs were stable after lyophilization, which caused no change in size (fig. S3). The ability to be stable after many freeze-thaw cycles as well as following lyophilization is a benefit of the tPNPs that is not possible with many other mRNA NP formulations.

### tPNPs are able to simultaneously generate and stimulate CAR T cells in vitro

**Fig. 3.**
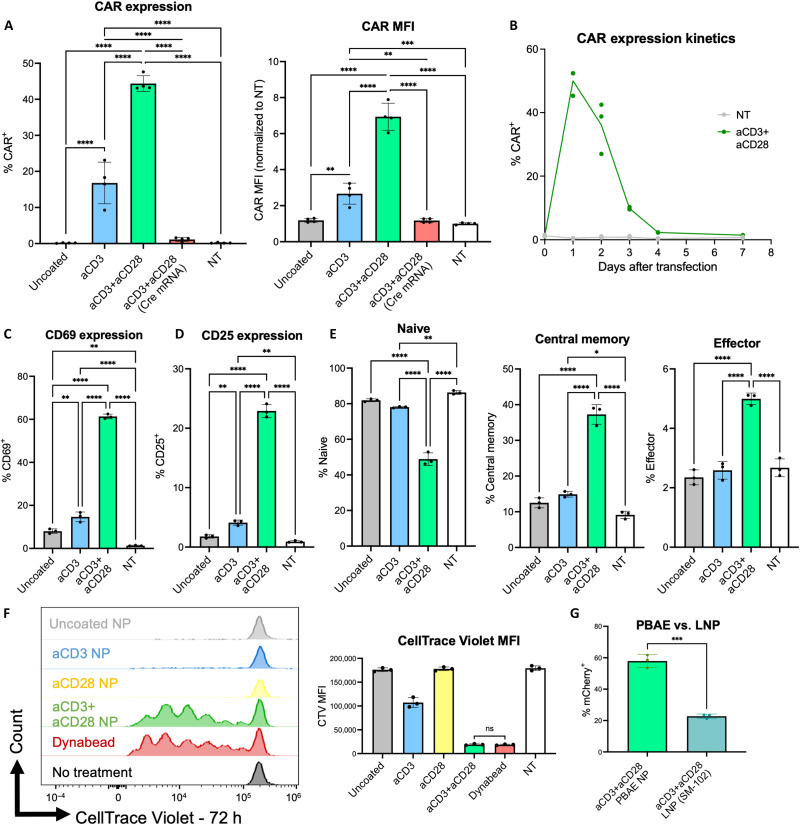
Incorporation of costimulatory targeting ligands improves transfection and enables robust T cell stimulation and transfection with CAR-encoding mRNA in vitro. (**A**) Primary murine T cell transfection was evaluated with tPNPs in naïve cells at a dose of 100 ng of mRNA per 50,000 cells (*n* = 4). Transfection, as measured by expression of an anti-CD19 CAR construct and the mean fluorescent intensity (MFI) of that CAR expression, is shown. (**B**) CAR expression in primary murine T cells was analyzed over time. T cells were cocultured with tPNPs on day 0, and CAR expression was measured after 1, 2, 3, 4, and 7 days (*n* = 3). (**C** and **D**) T cell activation after 24 hours (h) of coculture with tPNPs was analyzed via CD69 (C) and CD25 (D) expression (*n* = 3). (**E**) T cell memory phenotype after 24 hours of coculture with tPNPs was analyzed via CD44 and CD62L expression (*n* = 3). Memory subsets were delineated as follows: Naïve: CD44-CD62L^+^, central memory–like: CD44^+^CD62L^+^, effector memory–like: CD44^+^CD62L^−^. (**F**) T cell proliferation after 72 hours of coculture with tPNPs was measured via CellTrace Violet (CTV) signal. Proliferation is indicated by the number of peaks in CTV signal, with each peak representing a cell division from the original parent population. Proliferation was evaluated relative to a no treatment negative control and a Dynabead positive control, which is a commercially used reagent for T cell stimulation. (**G**) T cell transfection with aCD3+aCD28 PBAE NPs was compared to transfection with aCD3+aCD28 targeted LNPs using the Moderna LNP/SM-102 ionizable lipid formulation at a dose of 100 ng of mRNA per well of a 96-well plate. Statistical analysis for all experiments was performed using a one-way analysis of variance (ANOVA) with Tukey’s multiple comparisons test, *P < 0.05, **P < 0.01, ***P < 0.001, and ****P < 0.0001. ns, not significant.

We evaluated the ability of these particles to simultaneously stimulate and transfect primary murine T cells in vitro. We formed tPNPs with mRNA encoding a second generation anti-CD19 CAR (fig. S4) at a polymer/mRNA weight/weight ratio of 40 (fig. S5). Naïve murine T cells were isolated and incubated with tPNPs at a dose of 100 ng mRNA per well of a 96-well plate for 24 hours. tPNPs demonstrated CAR transfection and expression in a ligand and CAR mRNA–mediated manner (Fig. 3A), with particles functionalized with aCD3+aCD28 demonstrating higher CAR expression (44 ± 1%) than tPNPs with aCD3 alone (17 ± 3%). This is consistent with previously reported findings, as ex vivo CAR T cell engineering practice often requires T cell stimulation with both aCD3 and aCD28 to enhance viral transduction (*8*). The base, uncoated formulation demonstrated little to no T cell transfection, further highlighting the necessity of incorporating T cell–specific ligands into the NP for in vitro transfection. CAR expression for all tPNPs was compared to a nontreated (NT) control and noncoding aCD3+aCD28 Cre mRNA control. CAR expression was transient, with transfection peaking in T cells 1 day after NP incubation and returning to baseline levels by day 7 (Fig. 3B). In addition, treatment of T cells with aCD3 or aCD3+aCD28 tPNPs led to a decrease in the percent of cells that stained for being CD3 positive in vitro via flow cytometry (fig. S6). Despite this change in CD3 staining, there was no change in CD4 or CD8 expression between the uncoated and coated NPs. In addition, this trend in decreased CD3 staining but not CD4 or CD8 staining was observed when T cells were treated with sodium azide to inhibit uptake before the addition of tPNPs in vitro. From this, we concluded that the decrease in CD3 expression was not due to T cell receptor (TCR) internalization and recycling after NP binding to CD3 but was most likely due to competition of aCD3 antibodies on tPNPs with CD3 staining sites on the T cell for flow cytometry. These findings informed future flow cytometric analysis of tPNP transfection in vitro and in vivo, whereby we gated on CD4 and CD8 as T cell markers, rather than CD3.

T cell activation phenotype was analyzed 24 hours after coincubation with tPNPs. Stimulation with aCD3+aCD28 NPs demonstrated significantly higher expression of activation markers CD25 and CD69 than stimulation with aCD3 or uncoated NPs (Fig. 3, C and D). Analysis of T cell memory after 24 hours was consistent with activation marker expression, as cells stimulated with aCD3+aCD28 tPNPs demonstrated significantly greater proportions of “central memory–like” (CD44^+^CD62L^+^) and “effector-like” (CD44^+^CD62^−^) phenotype and down-regulated naïve phenotype compared to other NP conditions (Fig. 3E). To further characterize the role of ligand presentation in stimulating T cells, primary T cells were labeled with CellTrace Violet and incubated with tPNPs for 3 days. In addition to the previously examined uncoated, aCD3, and aCD3+aCD28 formulations, T cell proliferation was compared to an aCD28-only tPNP and to commercially sold aCD3+aCD28 Dynabeads as a positive stimulation control (*51*). While the uncoated and aCD28 stimulation groups did not elicit any T cell proliferation, the aCD3 alone tPNP did elicit some weak proliferation, with most T cells having not undergone any divisions (Fig. 3F). Combining aCD3+aCD28 onto the same tPNP elicited robust T cell stimulation comparable to proliferation caused by a Dynabead. These results highlight the importance of presenting ligands against both the TCR and a costimulatory ligand for full T cell stimulation and demonstrate the ability of this platform to stimulate T cells comparably to commercially used materials. In addition, the transfection efficiency of aCD3+aCD28-conjugated PBAE NPs was compared to aCD3+aCD28-conjugated Moderna LNPs using the SM-102 ionizable lipid as these LNPs represent a comparable, FDA-approved mRNA delivery standard (Fig. 3G) (*14*). The targeted PBAE NPs demonstrated enhanced transfection of primary murine T cells compared to the Moderna-based targeted LNPs, highlighting the efficacy of this polymeric platform for T cell transfection. Ultimately, these experiments outline the development and in vitro characterization of a targeted polymeric platform that is capable of robust T cell transfection and stimulation when functionalized with signals against CD3 and CD28.

### tPNPs demonstrate altered spleen-specific tropism in vivo

**Fig. 4.**
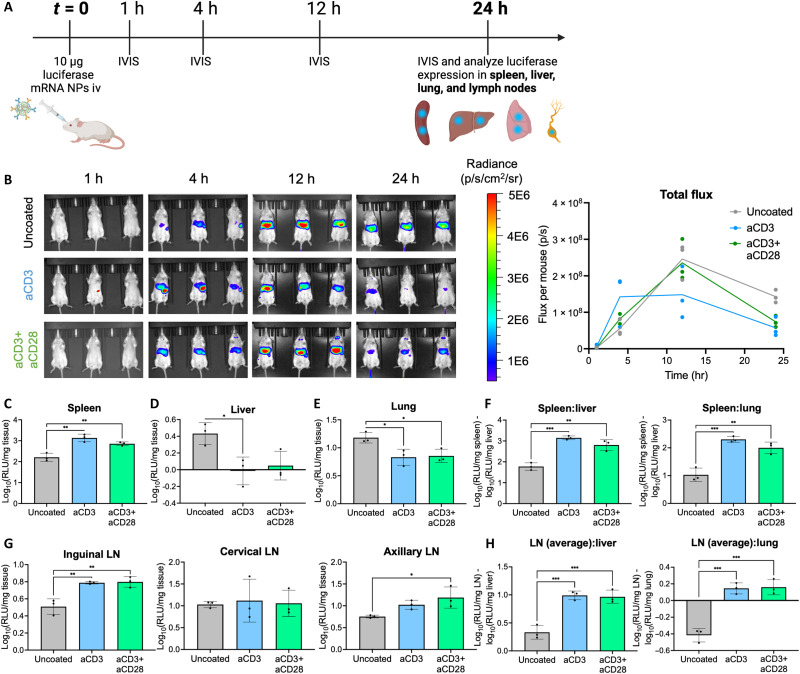
Incorporation of T cell ligands alters tropism toward lymphoid organ transfection in vivo. (**A**) Schematic of in vivo biodistribution study. Ten micrograms of tPNPs delivering luciferase-encoding mRNA was administered intravenously (iv) to albino C57BL/6 mice (*n* = 3). Whole-mouse luminescent signal was acquired via IVIS 1, 4, 12, and 24 hours after administration. (**B**) Whole-mouse luminescent images and total signal acquired at each time point. hr, hours. (**C**) After 24 hours, the mice were euthanized and spleens, livers, lungs, inguinal LNs, cervical LNs, and axillary LNs were isolated, weighed, processed, and analyzed for luciferase expression per milligram of tissue. Luminescent signal was up-regulated in the spleen in tPNP-treated mice relative to the uncoated NP group. Luminescent signal was analyzed in the (**D**) liver and (**E**) lung, with tPNP groups demonstrating lower transfection relative to the uncoated NP. (**F**) NP spleen tropism relative to the liver and lung was determined by subtracting the spleen luminescence from the liver or lung luminescence, as all values are reported as log_10_. (**G**) Luminescent signal was analyzed in the inguinal, cervical, and axillary LNs, with tPNPs demonstrated increased transfection in the inguinal and axillary LNs. (**H**) NP LN tropism relative to the liver and lung was determined by subtracting the average luminescence across all three LNs from the liver or lung luminescence. All bioluminescent signals per milligram of tissue were log normalized. Statistical analysis for each individual organ luminescence was performed using a one-way ANOVA with Tukey’s multiple comparisons test, *P < 0.05, **P < 0.01, and ***P < 0.001.

After validating the functionality of our tPNP platform in vitro, we next studied the transfection kinetics and efficiency of these particles in vivo. tPNPs delivering luciferase-encoding mRNA were administered intravenously at a dose of 10 μg mRNA per mouse, and whole-body transfection was measured at 1, 4, 12, and 24 hours after particle delivery via in vivo luminescence imaging (IVIS) (Fig. 4A). In addition, after 24 hours, mouse spleens, livers, lungs, inguinal lymph nodes (LNs), cervical LNs, and axillary LNs were isolated, lysed, homogenized, and analyzed for luciferase expression. While there was minimal luminescent signal 1 hour after NP delivery, total expression across all coated and uncoated tPNP group in the thoracic cavity increased after 4 hours and peaked at 12 hours (Fig. 4B).

After 24 hours, total luciferase signal was higher in the uncoated NP group compared to the aCD3 and aCD3+aCD28 tPNP groups. While at first, this whole-body signal may suggest that tPNPs are less effective than their untargeted counterparts, analysis of transfection on the organ scale reveals that transfection is higher in the spleen with aCD3 and aCD3+aCD28 tPNPs (Fig. 4C) and lower in the liver and lungs (Fig. 4, D and E). Given that these three organs are often associated with NP systemic delivery (*52*), the decreased whole-body luminescent signal from tPNPs can most likely be attributed to the decrease in luciferase signal seen in the liver and lungs. In the context of in vivo T cell engineering, NP delivery to lymphoid organs like the spleen is advantageous as this is the largest secondary lymphoid organ in the body and a major reservoir of T cells (*53*). Ultimately, analyzing the ratio of luminescent signal in the spleen relative to that in the liver or lungs reveals that the incorporation of a targeting ligand onto the base NP modifies tropism toward the spleen and away from the liver and lungs (Fig. 4F).

Historically, NP delivery to LNs is challenging as these organs have anatomical and structural components that tend to clear or exclude foreign nanosized cargo (*54*, *55*). We observed higher luciferase signal in the inguinal and axillary LNs (Fig. 4G). Analyzing the average luminescence signal across the three LNs relative to the signal in the liver and lungs reveals that tPNP formulations alter tropism toward these LNs and away from the liver and lungs in a manner similar to the spleen (Fig. 4H). In addition, no ligand-dependent transfection was observed in the heart, and a modest (0.4 RLU/mg tissue), but statistically significant increase in kidney transfection was observed by the aCD3+aCD28 tPNP relative to the uncoated NP (fig. S7). Moreover, the total raw luminescence values for each organ can be found in fig. S8. Ultimately, these findings regarding whole body– and organ-level transfection support the finding that tPNPs, with unique chemical composition compared to LNPs, can modify the tropism of an NP toward lymphoid organs and decrease transfection in nonlymphoid organs.

### tPNPs demonstrate altered T cell–specific tropism in vivo

**Fig. 5.**
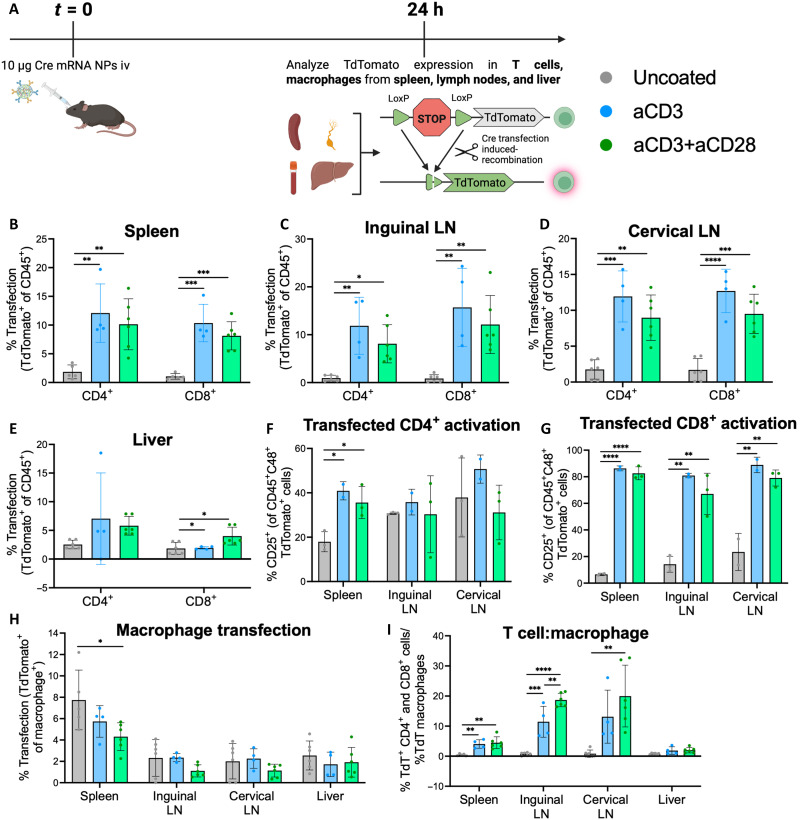
T cell–targeted NPs demonstrate robust T cell transfection and selectivity in vivo. (**A**) Schematic of in vivo cellular transfection study. Ten micrograms of tPNPs delivering Cre-encoding mRNA was administered intravenously to Ai9 mice (*n* = 4 to 6). After 24 hours, the mice were euthanized and spleens, inguinal LNs, cervical LNs, livers, and bloods were isolated, processed, and analyzed for transfection TdTomato signal in T cells (CD4^+^ or CD8^+^), and macrophages (Ly6G lo, F4/80^+^CD11b^+^) via flow cytometry. (**B** to **D**) T cell transfection was analyzed in secondary lymphoid organs—spleen (B), inguinal LN (C), and cervical LN (D). (**E**) T cell transfection was also analyzed in the liver. (**F** and **G**) Activation of transfected T cells was analyzed via CD25 expression in the CD4^+^TdTomato^+^ (F) or CD8^+^TdTomato^+^ (G) population within each lymphoid organ. (**H**) Macrophage transfection was also evaluated across the spleen, LNs, and liver. (**I**) T cell selectivity across all organs was determined by dividing the total cumulative % of TdTomato^+^ CD4 and CD8 cells by the total % of TdTomato^+^ macrophages, with all tPNPs demonstrating increased T cell:macrophage selectivity in lymphoid organs relative to the untargeted NPs. For (B) and (E), individual statistical analysis for transfection of CD4^+^ or CD8^+^ cells in each organ was performed using a one-way ANOVA with Tukey’s multiple comparisons test. In (F) and (G), individual statistical analysis of CD25 expression in transfected CD4^+^ or CD8^+^ cells for each organ was performed using a one-way ANOVA with Tukey’s multiple comparisons test. In (H), individual statistical analysis of transfection of macrophages in each organ was performed using a one-way ANOVA with Tukey’s multiple comparisons test. In (I), individual statistical analysis of T cell:macrophage selectivity in each organ was performed using a one-way ANOVA with Tukey’s multiple comparisons test, *P < 0.05, **P < 0.01, ***P < 0.001, and ****P < 0.0001.

To characterize the cell type–specific transfection of tPNPs, the Ai9 mouse model was used, whereby successful transfection with Cre-encoding mRNA causes the cell to undergo Cre-Lox recombination at the genomic level and stably express a TdTomato reporter protein for sensitive detection of transfection at the single-cell level. tPNPs were administered to Ai9 mice at a dose of 10 μg of Cre mRNA per mouse. Spleens, inguinal LNs, cervical LNs, blood, and livers were harvested after 24 hours and analyzed for TdTomato expression in T cells and macrophages (Fig. 5A). T cell transfection for in vivo samples was analyzed as a subset of CD4^+^ and CD8^+^ T cells. Analysis of CD4 and CD8 expression from Ai9 mice treated with tPNPs revealed some T cell depletion occurring in the LNs, as measured by the decrease in the percent of CD4^+^ and CD8^+^ T cells (as a subset of all CD45^+^ immune cells) in samples treated with tPNPs compared to those treated with the uncoated NP group (fig. S9). This phenomenon of T cell depletion via aCD3-targeted NPs has previously been reported by other groups and is a common phenomenon associated with systemic administration of the 145-2c11 clone of the aCD3 monoclonal antibody (*32*, *33*).

Robust genetic recombination-mediated CD4^+^ and CD8^+^ T cell transfection was observed across the three lymphoid organs analyzed (Fig. 5, B to D). In the spleen, inguinal LN, and cervical LN, tPNPs demonstrated significantly higher T cell transfection than the uncoated NPs. While splenic T cell transfection has previously been observed by other groups, the additional robust transfection observed in the inguinal and cervical LNs has previously not been reported, highlighting our platform. These findings further corroborate data observed from the organ-level luciferase analysis, where elevated transfection was observed in the LNs with the tPNPs (Fig. 4F). No ligand-dependent T cell transfection was observed in the liver (Fig. 5E), which is also consistent with findings from Fig. 4D, which show that total transfection in the liver is decreased with the incorporation of targeting ligands on the NP. The activation of transfected cells was also measured by analyzing CD25 expression. Up-regulated CD25 expression was observed in transfected CD4^+^ and CD8^+^ cells from the spleen, inguinal, and cervical LN treated with aCD3 and aCD3+aCD28 NPs (Fig. 5, F and G). Notably, the transfected CD8^+^ T cells (CD8^+^TdTomato^+^) in the uncoated NP treatment group demonstrated lower CD25 expression than the transfected CD8^+^ cells in the aCD3 and aCD3+aCD28 groups. These data suggest that the T cell activation observed in vivo is driven by T cell stimulation with ligand and not successful transfection. These results are consistent with the in vitro findings (Fig. 3) showing that tPNPs that demonstrated higher T cell transfection also demonstrated more robust activation. In vivo targeting with aCD3 NPs elicited CD25 expression that was similar to stimulation with aCD3+aCD28 NPs, although this trend was not observed in vitro. Anti-CD3 monoclonal antibodies alone have been demonstrated to stimulate T cells in vivo, and further characterization of T cell function and phenotype for aCD3 tPNP–treated mice is necessary (*33*). Ultimately, these results highlight the capability of tPNPs to transfect and stimulate T cells in vivo.

Transfection of macrophages across the different organs was also analyzed as macrophages are a common phagocytic cell responsible for the uptake of systemically delivered NPs. Mice treated with tPNPs demonstrated decreased macrophage transfection in the spleen, inguinal LN, and cervical LN compared to the baseline uncoated NPs, with aCD3+aCD28 NPs demonstrating lower transfection than mice treated with aCD3 NPs (Fig. 5H). Last, analyzing the ratio of the total percent of CD4^+^ and CD8^+^ T cells transfected in a treated mouse relative to the percent of macrophages transfected in the same mouse reveals that tPNPs exhibit significant T cell selectivity across all organs compared to uncoated NPs (Fig. 5I). In addition, aCD3+aCD28 NPs demonstrate higher selectivity than aCD3 NPs, highlighting the increased on-target transfection enabled by targeting with two ligands instead of one. This selectivity is most apparent in the inguinal and cervical LNs, where there is very low macrophage transfection by tPNPs (<1%). Moreover, this selectivity demonstrates the ability of active targeting with both aCD3 and aCD28 to minimize off-target transfection in macrophages, a cell type that would otherwise demonstrate higher particle uptake. Analysis of TdTomato signal in dendritic cells, B cells, natural killer (NK) cells, monocytes, and neutrophils revealed that there was no significant off-target transfection of these other cell types (fig. S10). Together, the whole organ luciferase transfection data shown in Fig. 4 and the cell-type Cre transfection shown in Fig. 5 highlight the ability of tPNPs to modulate particle tropism on the organ and cellular scale to minimize delivery to the liver and macrophages and increase delivery to lymphoid organs and T cells.

### CAR mRNA delivery via tPNPs elicits potent B cell depletion

**Fig. 6.**
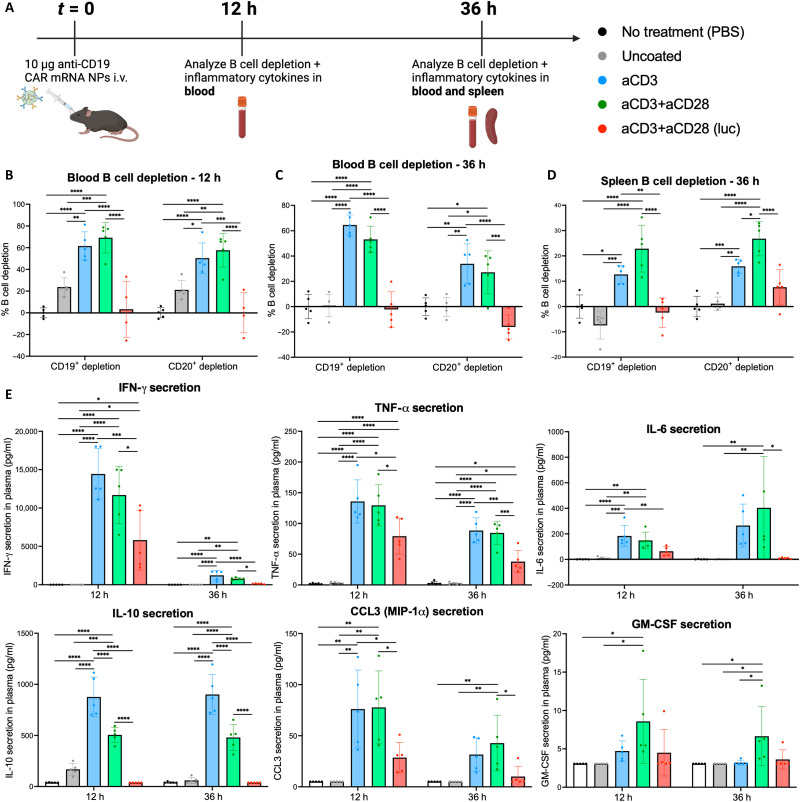
Systemic administration of CAR-encoding NPs enables robust B cell depletion in the blood and spleen. (**A**) Schematic of in vivo CAR-mediated B cell depletion study. Ten micrograms of tPNPs delivering anti-CD19 CAR-encoding mRNA was administered intravenously to C57BL/6 mice (*n* = 5). Mice were bled 12 and 36 hours after NP administration. B cell depletion was measured in blood cells and systemic cytokine secretion was measured in the plasma. Mice were euthanized at the 36-hour time point, and spleens were isolated and processed to measure B cell depletion. (**B** to **D**) B cell depletion was measured in the blood at 12 hours (B) and 36 hours (C), as well as the spleen at 36 hours (D). Significant CD19 and CD20 B cell killing was elicited by aCD3 and aCD3+aCD28 CAR tPNPs compared to uncoated CAR NPs and control aCD3+aCD28 luciferase mRNA NPs. (**E**) Systemic secretion of cytokines, IFN-γ, TNF-α, IL6, IL-10, CCL3, and granulocyte-macrophage colony-stimulating factor (GM-CSF), was measured in the plasma at 12 and 36 hours. For (B) to (D), individual statistical analysis for depletion of CD19^+^ or CD20^+^ cells in each organ was performed using a one-way ANOVA with Tukey’s multiple comparisons test. For (E), individual statistical analysis for secretion of each cytokine at the 12- or 36-hour time point was performed using a one-way ANOVA with Tukey’s multiple comparisons test, *P < 0.05, **P < 0.01, ***P < 0.001, and ****P < 0.0001.

After validating tPNP in vivo transfection and T cell tropism, tPNPs were used in a functional model of CAR-mediated B cell depletion. The same mRNA encoding for a second-generation anti-CD19 CAR construct with CD28 costimulatory domain used in the in vitro studies (Fig. 2) was used in all functional B cell depletion studies. B cell depletion was evaluated in the blood 12 and 36 hours after systemic administration of tPNPs delivering 10 μg of CAR mRNA per mouse (Fig. 6A). B cell depletion was also measured in the spleen 36 hours after tPNP injection. The spleen is the largest reservoir of B cells in the body, so depleting a significant portion of B cells in this organ would be a good indicator of the therapeutic potency of tPNPs (*53*, *56*). In addition to an NT saline control, B cell depletion with CAR tPNPs was also compared to an aCD3+aCD28 tPNP delivering nontherapeutic luciferase mRNA. B cell depletion was measured via changes in the percent of CD19^+^ and CD20^+^ cells among circulating leukocytes as both are common markers used to characterize developing and mature B lymphocytes, respectively (*57*).

B cell depletion was observed to depend on both the tPNP-targeting ligand and the CAR mRNA cargo. At the 12-hour time point in the blood, aCD3 and aCD3+aCD28 tPNPs elicited 62 ± 6% and 69 ± 6% CD19^+^ B cell depletion, respectively, with no depletion observed in the saline and luciferase mRNA tPNP control groups (Fig. 6B). B cell depletion was also observed in the untargeted CAR NP group, although this was significantly lower (24 ± 4%) than any of tPNP groups, highlighting the importance of T cell targeting for eliciting this therapeutic effect. By the 36-hour time point, B cell depletion in the blood was only observed in the tPNP groups (Fig. 6C). At both time points, lower CD20^+^ B cell depletion was observed relative to CD19^+^ depletion. This deeper depletion in the CD19^+^ B cell population is most likely due to the use of a CD19-targeted CAR mRNA construct. In the spleen at 36 hours, B cell depletion was only observed in the tPNP groups, with aCD3+aCD28 NPs demonstrating higher B cell depletion (23 ± 4%) than aCD3 NPs (13 ± 2%) (Fig. 6D). Although not a commonly used clinical readout, this depletion of splenic B cells 36 hours after NP delivery highlights the ability of tPNPs to elicit a strong therapeutic effect within tissues as well as in circulation.

Serum cytokines present in the blood were measured 12 and 36 hours after treatment injection (Fig. 6E). Specifically, circulating levels of interferon-γ (IFN-γ), tumor necrosis factor–α (TNF-α), CCL3, CCL4, interleukin-6 (IL-6), and granulocyte-macrophage colony-stimulating factor were measured as these cytokines are frequently associated with CAR-mediated target cell killing in the clinic. Secretion of all inflammatory cytokines was highest in CAR-tPNP groups, which further corroborates the potent B cell depletion elicited by these treatments. While cytokine levels in the luciferase aCD3+aCD28 tPNP were lower than the aCD3+aCD28 CAR tPNPs, they were elevated relative to the uncoated CAR NPs and PBS control, suggesting that the administration of the antibody-NP elicits some systemic cytokine production regardless of mRNA cargo. Some cytokines, like TNF-α and IL-6, achieved long-lasting durable secretion across the 36 hours, while others like IFN-γ and CCL4 peaked at 12 hours and returned to low or baseline levels by 36 hours. Ultimately, these data outline the ability of tPNPs to elicit potent in vivo B cell depletion in a CAR mRNA and targeting ligand-mediated manner.

### Characterization and optimization of CAR tPNP–mediated therapeutic efficacy

**Fig. 7.**
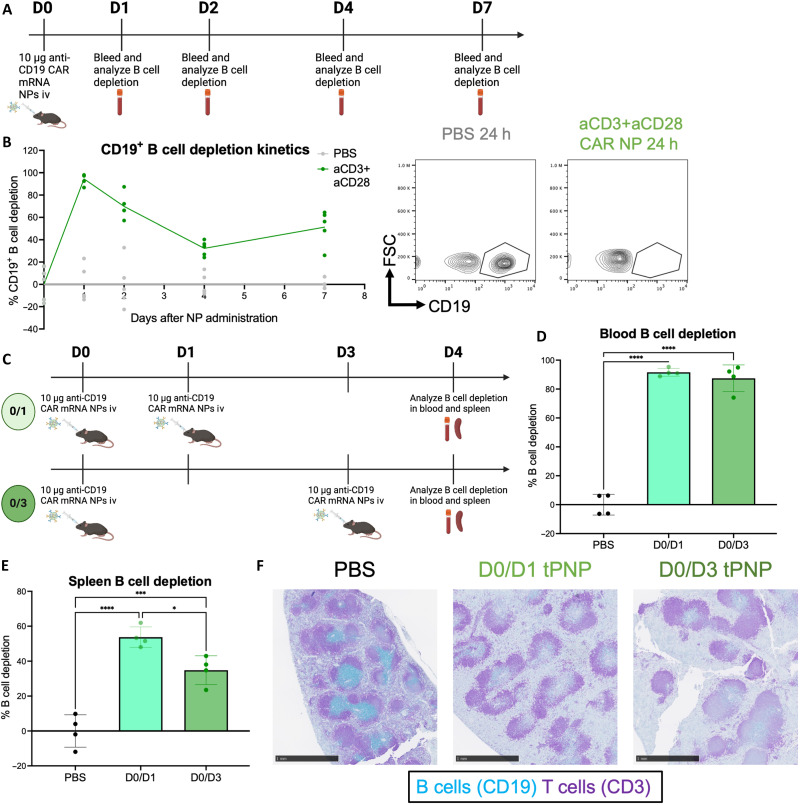
Characterization of tPNP-mediated in vivo B cell depletion kinetics and repeated dosing. (**A**) Schematic of in vivo CAR-mediated B cell depletion kinetics study. Ten micrograms of tPNPs delivering anti-CD19 CAR-encoding mRNA was administered intravenously to C57BL/6 mice (*n* = 5). Mice were bled on day 0 before tPNP administration to establish a baseline level of B cells in the blood. After aCD3+aCD28 CAR tPNP administration, the mice were bled on days 1, 2, 4, and 7, with B cell depletion being measured in blood cells at each time point. (**B**) B cell depletion was found to be transient, with depletion peaking on day 1 [(B) right] and decreasing over time. (**C**) Schematic of in vivo multiple CAR-tPNP dosing B cell depletion study. Ten micrograms of tPNPs delivering anti-CD19 CAR-encoding mRNA was administered intravenously to C57BL/6 mice (*n* = 4) on either days 0 and 1 or on days 0 and 3. Mice blood and spleens were harvested on day 4, and B cell depletion was compared to a baseline PBS-treated control mouse. (**D**) Potent B cell depletion was observed in the peripheral blood and (**E**) the spleen on D4. (**F**) Representative immunohistochemistry (IHC) stains of spleens treated with tPNPs across different dosing regimens. B cells are stained in blue, and T cells are stained in purple. All IHC stains are shown at 2.5× magnification; scale bar, 1 mm, and all samples can be found in fig. S12. For (D) and (E), statistical analysis for depletion of CD19^+^ B cells in the blood and spleen, respectively, was performed using a one-way ANOVA with Tukey’s multiple comparisons test, *P < 0.05, ***P < 0.001, and ****P < 0.0001.

Having demonstrated that tPNPs can elicit B cell depletion through targeted delivery of CAR mRNA, we next characterized the kinetics of this B cell depletion over time after a single injection of aCD3+aCD28 CAR tPNPs (Fig. 7A). We hypothesized that the B cell depletion effect would ablate over time, as in vitro characterization of CAR mRNA transfection in T cells (Fig. 3B) demonstrated that the CAR expression was transient and peaked at 24 hours. This trend was also observed in vivo, with B cell depletion in the blood reaching peak levels (95 ± 2%) 24 hours after CAR tPNP injection (Fig. 7B). At this 24-hour time point, almost no circulating B cells could be detected in the blood of treated mice, highlighting the tPNP’s potency shortly after administration (Fig. 7B, right). Unlike the in vitro CAR expression, which eventually returned to baseline levels by day 7, there was not full reconstitution of B cells in vivo by day 7. Instead, B cell depletion levels decreased to 32 ± 3% depletion by day 4 and stayed near that resting level by day 7 (51 ± 7%). Moreover, when comparing the in vivo kinetics of B cell depletion after a single dose of aCD3 or aCD3+aCD28 CAR tPNPs, significantly higher B cell depletion was observed at the day 14 time point with the aCD3+aCD28 tPNP (fig. S11). Although the level of B cell depletion observed in the blood did not significantly differ at the day 1, 4, or 7 time points between the aCD3 and aCD3+aCD28 tPNPs, this more potent, durable depletion observed with the aCD3+aCD28 tPNPs at day 14 highlights the value of using the dual-targeting approach for in vivo T cell engineering. These findings, combined with the observation that aCD3+aCD28 tPNPs demonstrated the highest T cell to macrophage transfection ratio (Fig. 5I), informed our decision to use aCD3+aCD28 for our final CAR tPNP redosing experiment.

On the basis of this understanding of B cell depletion kinetics, the therapeutic effect of dosing CAR-tPNPs multiple times was evaluated. Two dosing strategies were compared, where aCD3+aCD28 tPNPs were injected on days 0 and 1 or days 0 and 3, and B cell depletion was analyzed on day 4 (Fig. 7C). The dosing strategies did not result in observable differences in the levels of target cells in the blood as both D0/D1 and D0/D3 administration resulted in more than 85% B cell depletion by D4 (Fig. 7D). Higher B cell depletion in the spleen was observed with D0/D1 tPNP dosing compared to D0/D3 dosing (Fig. 7E). While this result was unexpected because of the transient nature of tPNP-mediated B cell depletion, the greater depletion in the spleen with D0/D1 injections compared to D0/D3 could result from the second injection on D1 inducing B cell depletion in an already depleted population. On the other hand, having the second injection on D3 with the D0/D3 dosing regimen could allow for greater reconstitution of splenic B cells after the first injection. The potent CD19^+^ B cell depletion observed at D4 in the spleen with D0/D1 dosing (54 ± 7%) was also higher than the splenic depletion observed with a single aCD3+aCD28 tPNP injection after 36 hours (23 ± 9%) (Fig. 6D), highlighting the therapeutic benefit of repeated dosing. This B cell depletion observed in the spleen was further supported by immunohistochemistry stains of spleens of tPNP-treated mice across both dosing regimens (Fig. 7F). While healthy, PBS-treated spleens have a large abundance of B cells, stained in blue, tPNP-treated spleens have far fewer of those B cells. In addition to determining the optimal dosing strategy for long-lasting B cell depletion, these tests highlight that tPNPs could be tolerated across multiple injections, a significant advantage over viral delivery systems. Moreover, to further validate tPNP tolerability, aCD3+aCD28 particles were administered to healthy mice across multiple dosing regimens (day 1, days 0 and 1, or days 0 and 7 injection), and measurements of mouse weight and aspartate aminotransferase (AST) and alanine aminotransferase (ALT) liver enzymes in the blood plasma were analyzed at different time points (fig. S13A). No significant changes in serum AST activity were observed between the single tPNP dose, multiple tPNP doses, and no treatment groups (fig. S13B). Analysis of serum ALT level showed an increase in activity relative to the no treatment group immediately following tPNP injection, although this effect was found to be transient, with long-term ALT activity resetting to baseline levels by day 14 (fig. S13C). Furthermore, no changes in mouse weight were observed across all groups over the course of 14 days (fig. S13D). These findings outline the overall tolerability of this platform across multiple injections. Ultimately, these results serve to further characterize the therapeutic B cell killing driven by aCD3+aCD28 CAR tPNPs and highlight the potency of this platform to elicit deep target cell depletion in the peripheral blood and spleen.

## DISCUSSION

Given the significant logistical complexities and costs associated with ex vivo CAR T cell manufacturing and administration, in vivo generation of CAR T cells has the potential to make these therapies safer and more accessible. Moreover, the transformative potential of this approach has led to growth of several companies working in the field of targeted gene delivery to T cells (*58*, *59*). This upswing in attention highlights the need for discovery and characterization of next-generation NP formulations that can effectively generate CAR T cells in vivo. The modular nature of PBAE-mRNA nanotechnology enables simple synthesis and potential scalability of different gene delivery vectors that can potentially address this need. Here, we outline a targeted polymeric NP formulation that can achieve robust T cell transfection and therapeutic efficacy by drawing from recent advancements in both nonviral mRNA delivery platforms and synthetic T cell–stimulating platforms.

The PBAE used in the tPNP system draws on distinct advantages from both LNPs and PNPs, with both having proven efficacy as mRNA delivery vectors. Unlike LNPs, which generally have four to five individual component lipids, PBAE NPs are generally formulated through simple mixing of the single polymer and the nucleic acid cargo (*42*). Furthermore, PBAE NPs are capable of being kept in long-term frozen storage or being lyophilized while LNPs traditionally cannot (*60*). These differences in processing and formulation have large translational implications in the clinic where clinicians may need to and handle and administer NPs under various storage and supply chain conditions, ultimately informing our decision to use a PBAE-based NP. PBAEs are also attractive from a safety perspective as they rapidly degrade in water with a half-life on the order of hours and have also been shown safe in vivo in multiple settings and animal models (*61*, *62*). However, for ligand conjugation of the antibody to the PBAE NP, we drew on inspiration from previous targeted LNP literature, where reports have used a functional PEG-lipid that self inserts into the lipid membrane of the particle (*32*, *38*). To use this similar PEG-lipid postinsertion technique, we synthesized a PBAE where a majority (80%) of the polymer’s side chains were composed of an amine-containing lipophilic monomer, 1-dodecylamine. In addition to enabling conjugation of a ligand to the NP surface, incorporation of the PEG lipid is crucial for enhancing NP circulation time and minimizing clearance (*63*). Previous targeted PBAE approaches used electrostatic interactions for ligand incorporation to the NP, whereby antibodies were coated with anionic polyglutamic acid so that they would associate with the cationic PBAE NP surface (*31*, *37*, *44*). While effective, this approach does not directly conjugate the ligand to the NP surface, meaning that the antibody, which is much larger than the attached anionic polyelectrolyte that associates with the NP, can potentially dissociate from the NP in vivo. Furthermore, the use of electrostatic interactions may also affect polymer-nucleic acid binding and complicate manufacture. Conversely, this lipophilic PBAE-PEG conjugation approach is similar to the proven method used by many LNPs while retaining the distinct advantages of PNPs.

Another way our approach differs from currently used T cell–targeted mRNA NP approaches is through the use of dual targeting ligands, anti-CD3 and anti-CD28, to engineer T cells in vivo. Targeting with both ligands specifically overcomes the two key challenges with T cell transfection. First, the use of two ligands confers greater T cell selectivity than one ligand alone and lessens concerns about off-target NP delivery. Second, the specific use of CD3- and CD28-targeting antibodies enables T cell stimulation and overcomes the need for T cell activation before intracellular delivery, analogous to the use of bispecific T cell–engaging antibodies. These dual targeting components, combined with a next-generation lipophilic PBAE formulation, enable robust mRNA delivery to primary murine T cells in vitro and in vivo. In murine reporter models, we demonstrate that tPNPs elicit robust in vivo T cell transfection at levels equal to or higher than previously reported formulations at a relatively moderate dose (10 μg mRNA per mouse). Previous approaches using CD3-targeted mRNA NPs achieved 4 to 7% transfection of all CD3^+^ T cells in the peripheral blood and spleen (*32*, *33*). In comparison, we observe 10 to 20% CD4^+^ and CD8^+^ T cell transfection across the spleen and multiple LNs in an Ai9 mouse model. While other lipid and polymer-based approaches targeting ligands like CD4, CD5, and CD8 are also able to achieve comparable transfection of circulating or splenic T cells, these studies do not study or achieve robust transfection in the LN, further highlighting the importance of our approach.

While it is critical that our tPNP demonstrates increased on-target T cell transfection, another key consideration is the need for reducing off-target non–T cell transfection. Many studies have previously shown that incorporating T cell–targeting ligands alters NP organ-specific transfection away from the liver and toward the spleen, as is the case with our tPNP formulations (*31*). However, many of these studies lack extensive characterization of cell type–specific tropism within each organ. This characterization is important as targeted NP formulations that demonstrate robust on-target T cell transfection may not also necessarily demonstrate decreased off-target transfection in other cells that may take up NPs like macrophages and dendritic cells. While our base, uncoated NPs demonstrate 8% macrophage transfection in the spleen, the addition of targeting ligands significantly decreases this macrophage transfection to 6 and 4% in the aCD3 and aCD3+aCD28 tPNP groups, respectively. Macrophage transfection is also decreased in the LNs, where baseline macrophage transfection with the uncoated NP is already low. Ultimately, the in vivo organ-level transfection and cell-level transfection data highlights the ability of tPNPs to demonstrate enhanced on-target tropism and decreased off-target tropism toward lymphoid organs/T cells and away from the liver/macrophages. To our knowledge, this level of cell-type selectivity has not previously been demonstrated in other NP delivery systems, including targeted LNPs. While many of the targeting ligands used in similar LNP studies are the same as those used in this study, differences in chemistry between LNP and PBAE base formulations may underlie the reduced off-target transfection by our PBAE NPs.

Last, we sought to demonstrate that this selective targeting of T cells within lymphoid organs could generate a therapeutically relevant effect when delivering mRNA encoding an anti-CD19 CAR. For the functional readout, B cell depletion in healthy C57BL/6J mice was used. Anti-CD19 CAR T therapies are inherently designed to kill both healthy and cancerous B cells, and so this model enables a translationally relevant assessment of in vivo CAR T cell function. B cell depletion was observed in a ligand- and CAR mRNA–mediated manner, showing that targeted delivery of the CAR-encoding mRNA is critical for eliciting a therapeutic effect. While no significant B cell depletion was observed with the aCD3+aCD28 tPNPs delivering nontherapeutic Cre mRNA, up-regulated secretion of certain inflammatory cytokines in the plasma at 12 hours, namely IFN-γ TNF-α, CCL3, and CCL4, was observed. This transient effect has previously been observed with systemic administration of anti-CD3 monoclonal antibodies or targeted NPs. Future investigation regarding cytokine secretion–related toxicity due to both the targeting ligand and CAR T–driven B cell killing in vivo is needed (*32*, *64*). Despite this, we did confirm that aCD3+aCD28 tPNPs were capable of being dosed multiple times and eliciting potent B cell depletion in both the peripheral blood and the spleen. Moreover, more than 50% B cell depletion was observed in the spleen at day 4 after repeated tPNP injections on D0 and D1. This level of splenic depletion is higher than previously published in vivo CAR T approaches. The potent splenic depletion, combined with almost full depletion of all circulating B cells merely 24 hours after a single injection further highlights the efficacy of the tPNP system.

Ultimately, tPNPs are an innovative polymeric platform that is relatively inexpensive and simple to manufacture. Their potency as highly selective gene delivery vectors to T cells rivals or outperforms current LNP and PNP standards. Last, the efficacy of tPNPs has been characterized across several therapeutic experiments of in vivo CAR T–mediated B cell depletion. These findings highlight that tPNPs have broad potential to engineer endogenous T cells for treatment of disease. Future investigation of this platform in different B cell–based malignancies spanning both murine and human models of cancer and autoimmune diseases are currently underway.

## MATERIALS AND METHODS

### PBAE synthesis

A lipophilic-hydrophilic PBAE blend was synthesized following methods previously described in (*65*). The diacrylate monomer, bisphenol A glycerolate (1 glycerol/phenol) diacrylate (B7), was dissolved in dimethylformamide and reacted with a hydrophilic amine-sidechain monomer, 4-(2-Aminoethyl)morpholine (S90), and a lipophilic amine–side chain monomer, 1-dodecylamine (Sc12), for 48 hours at 90°C for 48 hours to form a base polymer. After 48 hours, an excess of an amine-endcap monomer, diethylenetriamine (E63) was reacted with the base polymer for 1 hour at room temperature. The final end-capped PBAE, 7-90, c12-63 (80%), was then precipitated into diethyl ether, dried under a vacuum for 48 hours, resuspended in anhydrous dimethyl sulfoxide, and stored at −20°C.

### mRNA

In vitro–transcribed mRNA constructs used in this study encoded for either green fluorescent protein (GFP), mCherry, firefly luciferase, Cre recombinase, or an anti-CD19 CAR. GFP, mCherry, luciferase, and Cre mRNA were all purchased from TriLink Biotechnologies. Anti-murine CD19 CAR mRNA was purchased from RNA Technologies.

### Targeted PBAE NP formulation

Base NPs were formulated at a 40:1 weight/weight ratio of PBAE to mRNA. mRNA was diluted into a solution of 1 mM sodium acetate and the PBAE was diluted in a solution of 90% ethanol and 10% 5 mM sodium acetate. A functional DMG-PEG-NHS (Avanti Polar Lipids) lipid was incorporated to the base NP via the post-insertion technique. NPs were allowed to complex for 10 min and were then purified via dialysis with a 50 kDa dialysis tube into 1 mM sodium acetate for 1 hour.

After initial formulation, the base NP was combined with antibody. Antibodies used for NP targeting were mouse anti-CD3 (BioXcell, clone 145-2c11) and anti-CD28 (BioXcell, clone 37.51). Antibody and NP were combined at a 1:1 mass ratio of DMG-PEG-NHS to total antibody. The total amount of antibody on the surface of the NP was kept consistent, meaning that aCD3 tPNPs had as much total protein incorporated as aCD3+aCD28 tPNPs. The antibodies used were not processed or reduced to avoid affecting the overall function of the antibody. Antibody was conjugated to the functional PEG-lipid inserted on the base NP via NHS-activated peptide combination with primary amines on the protein. The antibody and NP were mixed at room temperature for 1 hour and were then transferred to 4°C where they were allowed to react for an additional 2 hours. Following conjugation, excess, unreacted antibody was removed via overnight dialysis with a 1000-kDa dialysis tube into >1 liter of 1× PBS. After dialysis, finished tPNPs were stored at −80°C for up to 3 months and were used within three freeze-thaw cycles.

### Targeted LNP NP formulation

Base LNPs were formulated on the basis of the commercially approved Moderna LNP standard, whereby SM-102 (Broadpharm), 1,2-distearoyl-sn-glycero-3-phosphocholine (DSPC) (Avanti Polar Lipids), cholesterol (Sigma-Aldrich), and DMG-PEG-NHS (Avanti Polar Lipids) were diluted in ethanol at the following relative molar ratio of 50:10:38.5:1.5. mCherry mRNA was diluted in 25 mM magnesium acetate, and the lipid phase and mRNA phase were complexed to form the LNPs. After base LNP complexation, ligands were conjugated following the same protocol as PBAE NPs, whereby base NPs were purified via dialysis with a 50-kDa tube, incubated with aCD3 and aCD28 antibodies, and then purified via dialysis with a 1000-kDa tube into PBS.

### Targeted NP characterization

tPNP size was analyzed via DLS and TEM. For DLS, NPs were diluted in PBS at a 1 to 5 ratio and were analyzed using a Zetasizer Pro (Malvern Instruments, Malvern, UK) to determine particle hydrodynamic diameter (*z*-average) and poly-dispersity index. For TEM, NPs were dried on a mesh copper grid (Electron Microscopy Sciences; Hatfield, PA, USA) and imaged using a Hitachi 7600 transmission electron microscope. NP surface charge (zeta potential) was also characterized using the Zetasizer Pro by measuring electrophoretic mobility of the NPs diluted in 0.1× PBS.

### Primary T cell isolation

Primary murine T cells were isolated from adult C57BL/6 mice spleens and LNs. Isolated organs were macerated and filtered through 70-μm cell strainers twice. After filtration, T cells were isolated via negative selection with murine Pan-T cell (CD3^+^) isolation kits and magnetic columns from Miltenyi Biotech (Auburn, CA, USA).

For in vitro experiments, isolated T cells were cultured in murine T cell media (RPMI 1640 supplemented with l-glutamine, nonessential amino acids, vitamin solution, sodium pyruvate, β-mercaptoethanol, 10% fetal bovine serum, and ciprofloxacin. In addition, the media were supplemented with recombinant murine IL-2 at a final working concentration of 10 ng/ml.

### In vitro T cell transfection

Primary murine T cells were plated in *n* = 3 or *n* = 4 in 96-well round-bottom plates at a concentration of 50,000 T cells in 100 μl of media per well. After plating, T cells were treated with tPNPs at a dose of 100 ng mRNA per well. For all in vitro transfection and phenotype experiments shown in Fig. 3, tPNPs delivering anti-CD19 CAR-encoding mRNA were used. For additional transfection experiments, tPNPs delivering GFP- or mCherry-encoding mRNA were used. In vitro transfection was measured after 24 hours. For assessment of in vitro CAR expression kinetics shown in Fig. 3B, T cells were treated with tPNPs, and then a subset of treated cells was harvested and analyzed at days 1, 2, 3, 4, and 7.

Samples were prepared for flow cytometric analysis of CAR expression via centrifugation at 500*g* for 5 min and resuspension in 100 μl of PBS with a 1:1000 dilution of Live Dead-Near IR (Thermo Fisher Scientific, catalog no. L10119) and a 1:250 dilution of biotinylated mouse CD19 protein (Sino Biological, catalog no. 50510-M08H-B). After staining with this viability dye and CD19 protein for 20 min, the cells were centrifuged and washed with PBS three times and resuspended in 100 μl of PBS with 1:350 dilution of fluorescent-tagged streptavidin (BioLegend, catalog no. 405203). After staining with the fluorescent secondary for 15 min, the cells were washed and analyzed via flow cytometry for CAR expression (Attune NxT). For GFP or mCherry transfection, T cells were only stained for viability before analysis via flow cytometry. Standard gating was used, whereby doublet and dead cells were excluded and CAR or GFP expression was only analyzed on the subset of live cells. Gating of transfected cells was determined by the background signal from the NT control group (fig. S14).

### In vitro T cell phenotypic analysis

T cell phenotype was measured via flow cytometry 24 hours after treatment with CAR tPNPs. A similar sample preparation workflow for in vitro transfection analysis was followed, whereby samples were centrifuged and resuspended in 100 μl of PBS with a 1:1000 dilution of Live Dead-Near IR (Thermo Fisher Scientific, catalog no. L10119), 1:100 dilution of CD25 (BioLegend), 1:100 dilution of CD69 (BioLegend), 1:100 dilution of CD44 (BioLegend), and a 1:100 dilution of CD62L (BioLegend). Cells were stained with this antibody cocktail for 20 min, washed, and analyzed via flow cytometry. Standard gating for exclusion of doublet and dead cell populations was used, and all phenotypic markers were gated on the basis of a fluorescence minus one control sample. T cell memory phenotype was determined via CD44 and CD62L expression with CD44^−^CD62L^+^ cells being classified as naïve, CD44^+^CD62L^+^ cells being central memory–like, and CD44^+^CD62L^−^ cells being effector memory-like (fig. S15).

For analysis of T cell proliferation in vitro, cells were stained with CellTrace Violet proliferation dye (Thermo Fisher Scientific, catalog no. C34557) after T cell isolation and before plating. The cells were harvested 72 hours after treatment with tPNPs and stained with Live Dead-Near IR, as previously outlined above. After staining, proliferation was analyzed via flow cytometry, with the number and height of the peaks of CellTrace Violet signal being indicative of the number of cell divisions the parent population had undergone and the relative number of cells in each daughter population, respectively.

### Animal care

The C57BL/6 (B6) and Ai9 mice strains were used for all in vivo experiments were maintained under the guidelines set by the Johns Hopkins University Institutional Animal Care and Use Committee, study approval number MO24M284. Black B6 mice were used for all in vitro and in vivo studies, except for the in vivo transfection study with luciferase-encoding mRNA shown in Fig. 4, where albino B6 mice were used to enable imaging of whole-body transfection. All mice used for both in vitro T cell isolation and in vivo studies were between 8 to 12 weeks old. Both male and female mice were used.

### In vivo organ-level transfection assessment

For whole body and organ-level transfection analysis, 10 μg of tPNPs delivering firefly luciferase–encoding mRNA were administered via retro-orbital injection to albino B6 mice. After tPNP injection, the mice were imaged at 1-, 4-, 12-, and 24-hour time points via IVIS. Ten minutes before each IVIS time point, the mice were injected intraperitoneally with d-Luciferin, potassium salt, and total luminescent flux was acquired. After the 24-hour imaging time point, the mice were euthanized and the following organs were harvested: spleen, liver, lung, inguinal LN, axillary LN, cervical LN, heart, and kidney.

Organs were then weighed and processed to acquire luciferase signal per milligram of tissue for each harvested organ. After weighing, the organs were suspended in a lysis buffer (Promega, catalog no. E3971), with 10 ml of lysis buffer being added per each gram of tissue, and placed in an ultrasonic processor (Qsonica, catalog no. Q55A) to fully break down the tissue. After lysis and homogenization, the tissue was frozen at −80°C and thawed to fully release all luciferase inside the cells. After processing, luminescent signal was acquired via a plate reader for each processed organ and normalized to the mass of the respective organ. This approach, whereby organs were lysed and homogenized before luminescent signal acquisition, rather than capturing signal of whole organs via IVIS, was used to ensure that all cells were able to use oxygen for luminescence expression.

### In vivo cellular-level transfection assessment

For analysis of in vivo transfection on a cellular scale, the Ai9 mouse model was used. This transgenic mouse strain is on a B6 background and has every cell engineered to express a TdTomato reporter protein preceded by a stop codon that is flanked by LoxP promoters. Upon successful transfection with Cre mRNA in a cell of the Ai9 mice, that cell will undergo Cre-Lox recombination to remove the stop codon and enable visualization of the TdTomato reporter. For analysis of cellular transfection with tPNPs, 10 μg of tPNPs delivering Cre-encoding mRNA were administered via retro-orbital injection to Ai9 mice. All Ai9 mice used for this study were between 8 to 12 weeks old and included both male and female mice. Twenty-four hours after injection, the following organs were harvested: spleen, inguinal LN, cervical LN, liver, and blood.

Harvested organs were then macerated and treated with ACK lysing buffer to lyse red blood cells. After lysis, the organs were filtered through cell strainers and stained for the following markers: live/dead viability (Thermo Fisher Scientific, catalog no. L10119), CD45 (BioLegend, catalog no. 103139), CD4 (BioLegend, catalog no. 100437), CD8 (BioLegend, catalog no. 100712), CD25 (BioLegend, catalog no. 102015), NKp46 (BioLegend, catalog no. 137608), CD19 (BioLegend, catalog no. 115549), CD11b (BioLegend, catalog no. 101212), CD11c (BioLegend, catalog no. 117311), Ly6G (BioLegend, catalog no. 127628), and F4/80 (BioLegend, catalog no. 123114). All the cells were gated as a subset of live and CD45^+^ cells (all immune), with the following cell types differentiated as follows: helper T cell (CD4^+^), killer T cell (CD8^+^), activated T cell (CD4^+^/CD8^+^ CD25^+^), NK cell (NKp46^+^), B cell (CD19^+^), macrophage (Ly6G lo, F4/80^+^ CD11b^+^), dendritic cell (Ly6G lo, F4/80^−^ CD11b^−^ CD11c^+^), and neutrophil (Ly6G hi). Expression of these cell type markers, along with transfection via TdTomato expression, was analyzed on a flow cytometer. Gating strategy for each cell type and phenotypic markers can be seen in figs. S16 to S19.

### In vivo CAR mRNA delivery and B cell depletion

For in vivo therapeutic B cell depletion experiments, mRNA encoding a second-generation anti-CD19 CAR construct was used, whereby expression of this mRNA in T cells would result in successful on-target killing of endogenous B cells. All mice used in CAR mRNA B cell depletion experiments were between 8 to 10 weeks old. For characterization of CAR mRNA–mediated B cell depletion, 10 μg of tPNPs were administered via retro-orbital injection to black B6 mice. Following injection, ~50 μl of blood was collected at 12 and 36 hours via cheek bleed. Plasma was then isolated from blood samples via centrifugation and was stored −80°C. Levels of inflammatory cytokines were measured using the Legendplex assay kit, which was performed following the manufacturer’s instructions. At the 36-hour time point, the mice were euthanized and spleens were harvested as well. Blood and spleens were processed via maceration, treatment with ACK lysis buffer, and filtration through cell strainers. After processing, all samples were stained for viability, CD45, and B cell markers CD19 and CD20. Baseline B cell levels were calculated as the average percent of CD19^+^ or CD20^+^ cells (as a subset of CD45^+^ cells) in the saline-treated control group. B cell depletion was then calculated as the relative decrease from this baseline in the percent of CD19^+^ or CD20^+^ cells for a tPNP-treated mouse (fig. S20).

For characterization of in vivo B cell depletion kinetics over the course of a week, aCD3+aCD28 CAR-tPNPs were injected to B6 on day 0. Mice were then bled via cheek bleed on day 0 (preinjection), 1, 2, 4, and 7. Bloods were processed and stained for viability, CD45, and CD19 to assess B cell depletion over time.

For characterization of the therapeutic effect of administering multiple doses of tPNPs, aCD3+aCD28 CAR-tPNPs were injected on days 0 and 1 or days 0 and 3. Mice bloods and spleens were then collected. Spleens were split into two halves, with half of the spleen being fixed in formalin for histological analysis. The remaining spleen and blood samples were processed and stained for viability, CD45, and CD19 to assess B cell depletion between the two dosing regimens on day 4.

### Histology

Fixed spleens from mice treated with multiple CAR-tPNP doses or a saline control were sectioned and stained by hematoxylin and eosin. Fixed spleens were also sectioned and stained via immunohistochemistry for CD3 and CD19 expression. All histology was performed by the Johns Hopkins Oncology Tissue Services core.

### Statistical analysis

All graphs presented depict means, with error bars depicting the SEM. All comparisons between multiple groups were made using one-way analysis of variance (ANOVA) statistical tests with Tukey’s post hoc tests Statistics were performed using GraphPad Prism software version 10, and statistical significance is indicated as follows: *P < 0.05, **P < 0.01, ***P < 0.001, and ****P < 0.0001.
