# Supplementary material for: Biodegradable targeted polymeric mRNA nanoparticles enable in vivo CD19 CAR T cell generation and lead to B cell depletion
Source: Sci Adv. 2026 Mar 11;12(11):eadz1722. doi: 10.1126/sciadv.adz1722 (PMC12978253; doi:10.1126/sciadv.adz1722)
Supplement: Supplementary file 1 — Figs. S1 to S20 [file sciadv.adz1722_sm.pdf]

Science Advances
AAAS

Supplementary Materials for

Biodegradable targeted polymeric mRNA nanoparticles enable in vivo CD19 CAR T cell generation and lead to B cell depletion

Manav Jain *et al.*

Corresponding author: Jordan J. Green, green@jhu.edu; Jonathan P. Schneck, jschnec1@jhmi.edu; Stephany Y. Tzeng, stzeng1@jhmi.edu

Sci. Adv. **12**, eadz1722 (2026)
DOI: 10.1126/sciadv.adz1722

This PDF file includes:

Figs. S1 to S20

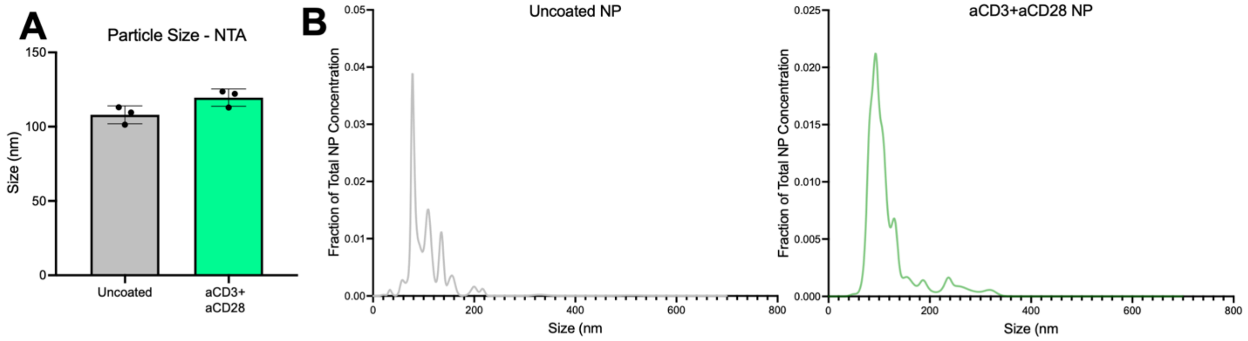

Figure S1. Characterization of tPNP size and distribution via nanoparticle tracking analysis (NTA). (A) tPNPs were diluted in PBS and a total of 1500 frames were measured over the course of 60 seconds for each sample via NTA. Average NP size was measured to be similar between the uncoated NP (108 ± 6 nm) and the aCD3+aCD28 tPNP (120 ± 6 nm). (B) Size distribution was also measured between the uncoated and aCD3+aCD28 tPNP."

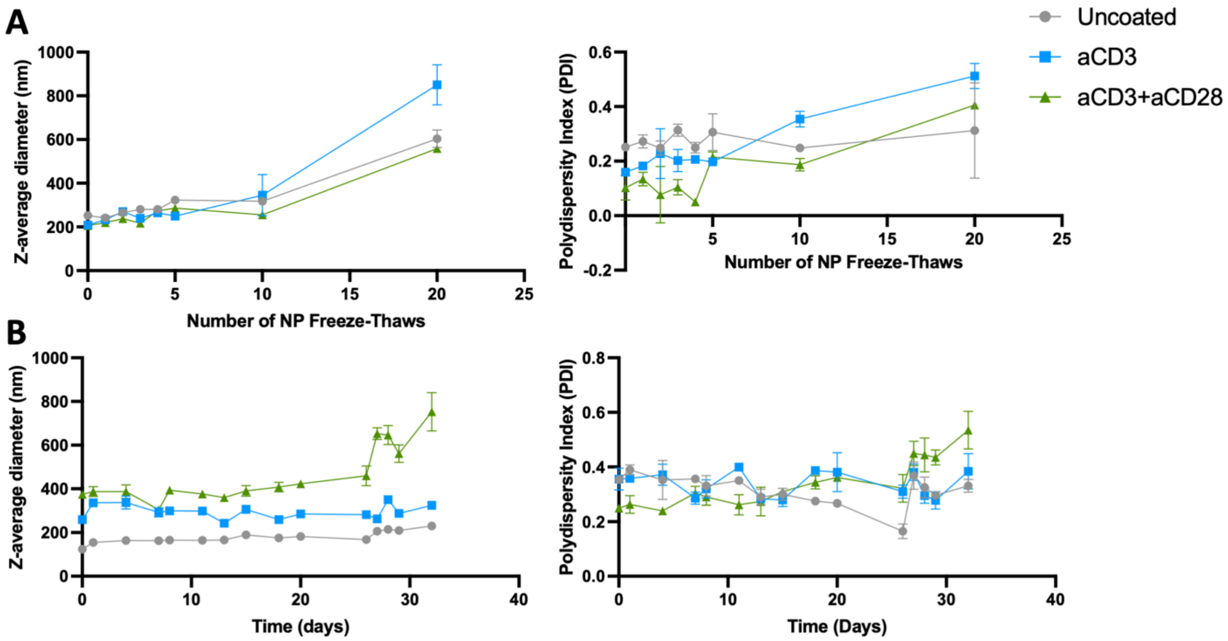

Figure S2. Characterization of tPNP size across freeze-thaw cycles and time. Uncoated, aCD3, and aCD3+aCD28 tPNPs were made and distributed across several aliquots. (A) Characterization of NP size across freeze-thaw cycles. Each tPNP aliquot for this study was frozen at -80ºC and thawed for the indicated number of freeze-thaw cycles. Afterwards, tPNP size and polydispersity (PDI) was measured using a Zetasizer. (B) Characterization of NP size over time. Each tPNP aliquot for this study was frozen at -80ºC and thawed on the indicated day. Afterwards, tPNP size and polydispersity (PDI) was measured using a Zetasizer.

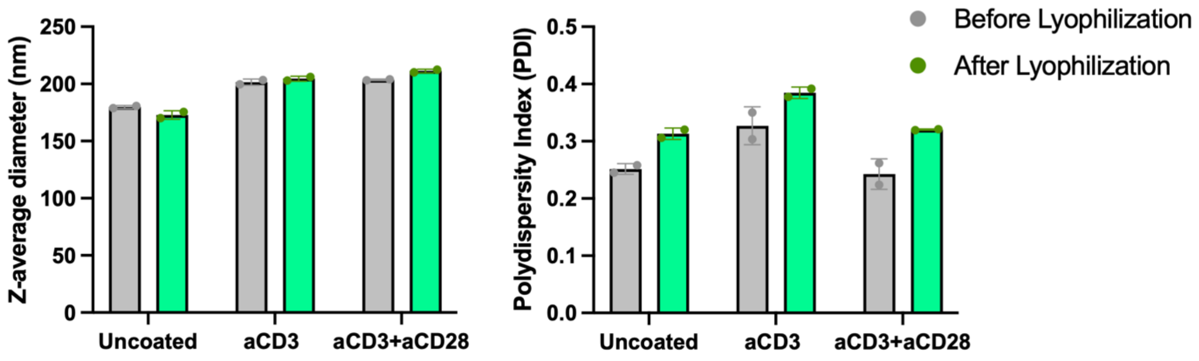

Figure S3. Characterization of tPNP size before and after lyophilization. tPNPs were formulated following the process outlined in Figure 2B and NP size and PDI was characterized using a Zetasizer. After formulation, sucrose was diluted in the NP solution as a cryoprotectant to reach a final concentration of 90 mg sucrose/mL. tPNPs were then frozen at -80ºC, removed, and lyophilized overnight. Following lyophilization, NPs were reconstituted in water and size and PDI was characterized using a Zetasizer.

Figure S4. Amino acid sequence for anti-CD19 chimeric antigen receptor (mCD19 1D3) mRNA construct

Label	Sequence
Signal Peptide	MGVPTQLLGLLLLWITDAIC
mCD19(1D3) VL	DIQMTQSPASLSTSLGETVTIQCQASEDIYSGLAWYQQKPGKSPQLLIY GASDLQDGVPSRFSGSGSGTQYSLKITSMQTEDEGVYFCQQGLTYPRT FGGGTKLELK
G4S Linker	GGGGS
G4S Linker	GGGGS
G4S Linker	GGGGS
mCD19(1D3) VH	VQLQQSGAELVRPGTSVKLSCKVSGDTITFYYMHFVKQRPGQGLEWI GRIDPEDESTKYSEKFKNKATLTADTSSNTAYLKLSSLTSEDTATYFCI YGGYYFDYWGQGVMVTVSS
CD28_MOUSE	IEFMYPPPYLDNERSNGTIIHIKEKHLCHTQSSPKLFWALVVVAGVLFC YGLLVTVALCVIWTNSRRNRGGQSDYMNMTPRRPGLTRKPYQPYAPA RDFAAYRP
CD28_MOUSE Hinge	IEFMYPPPYLDNERSNGTIIHIKEKHLCHTQSSPKL
CD28_MOUSE Transmembrane	FWALVVVAGVLFCYGLLVTVALCVIWT
CD28_MOUSE Intracellular	NSRRNRGGQSDYMNMTPRRPGLTRKPYQPYAPARDFAAYRP
CD3Z_MOUSE	RAKFSRSAETAANLQDPNQLFNELNLGRREEFDVLEKKRARDPEMGG KQQRRRNPQEGVYNALQKDKMAEAYSEIGTKGERRRGKGHDGLFQG LSTATKDTFDALHMQTLAPR

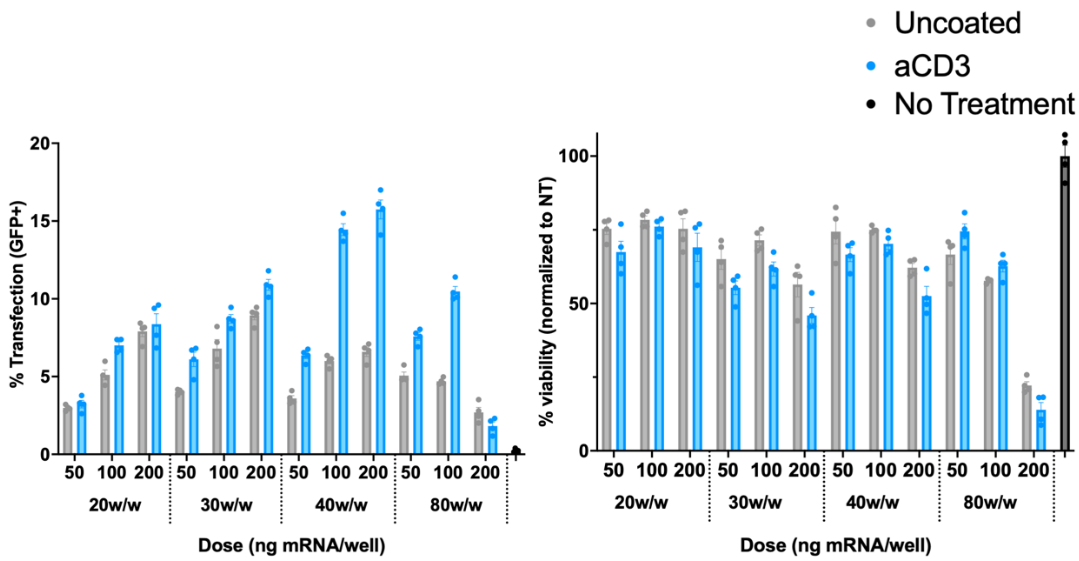

Figure S5. ***In vitro*** **transfection with aCD3 tPNPs reveals a 40 weight/weight ratio of polymer to mRNA to be optimal.** tPNPs were formulated following the process outlined in Figure 2B, with the weight/weight ratio of PBAE polymer to GFP-encoding mRNA being varied from 20 w/w to 80 w/w and all NPs being either uncoated or aCD3-coated. tPNPs were then co-incubated with primary naïve murine T cells, and T cell transfection and viability was measured after 24 hours.

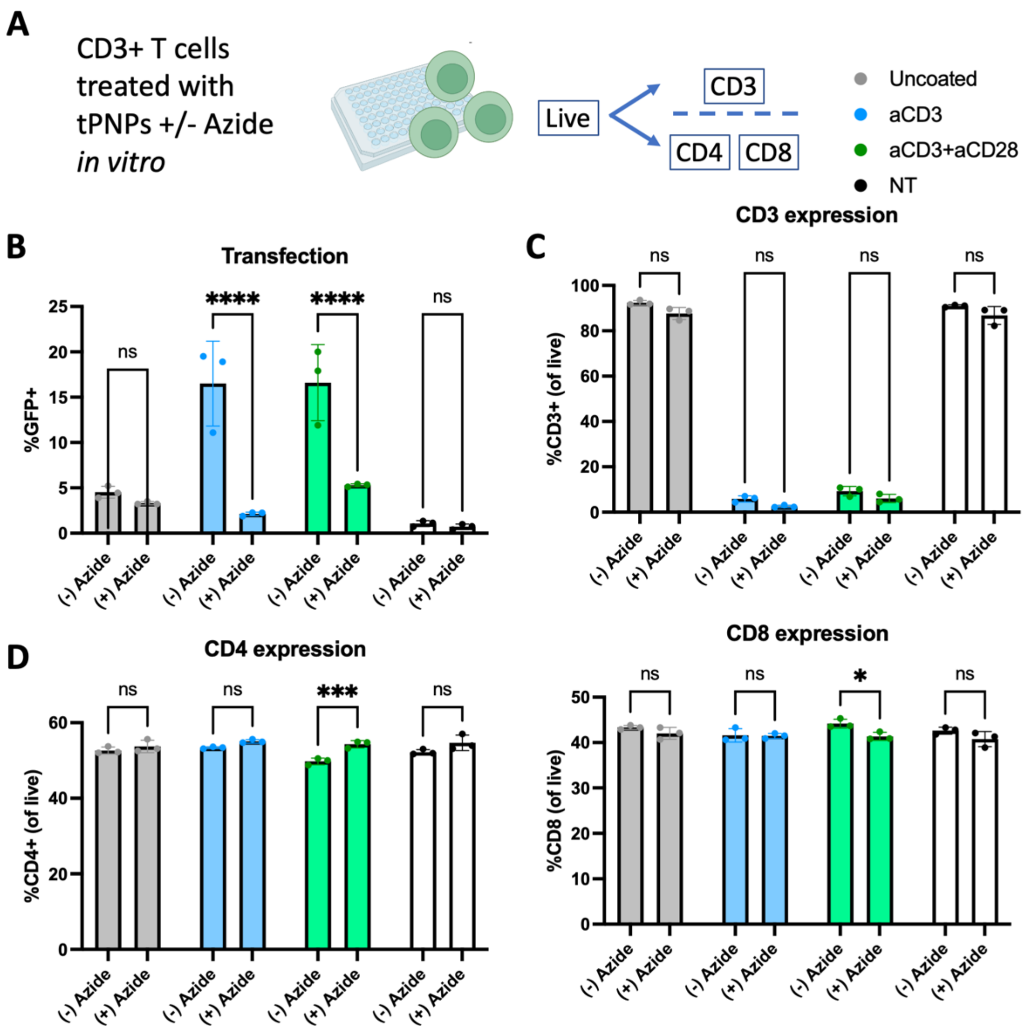

Figure S6. Treatment of isolated murine T cells with tPNPs decreases CD3 expression, but not CD4 or CD8 expression, *in vitro.* (A) Primary naïve murine T cells were co-incubated with uncoated, aCD3, or aCD3+aCD28 tPNPs delivering GFP-encoding mRNA. T cells were also treated with or without azide, which would inhibit tPNP uptake and internalization. After treatment for 24 hours, GFP transfection was measured via flow cytometry. T cells were stained for CD3, CD4, and CD8 expression, which was measured via flow cytometry. (B) tPNPs demonstrated higher GFP transfection than uncoated NPs, but the addition of azide diminishes this transfection. (C) T cell treatment with aCD3 and aCD3+aCD28 tPNPs demonstrates drastically reduced CD3 staining expression relative to the no treatment or uncoated NP control. Addition of azide does not affect the level of CD3 staining, suggesting that the decrease in CD3 staining is not due to NP internalization. (D) T cell treatment with aCD3 and aCD3+aCD28 tPNPs has not effect on CD4 and CD8 staining relative to the no treatment or uncoated NP control. Statistical analysis was performed using a one-way ANOVA with Tukey's multiple comparisons test. Comparisons between the with/without azide treatment groups are displayed for each nanoparticle condition. $^{*}P < 0.05$, $^{***}P < 0.001$, and $^{****}P < 0.0001$.

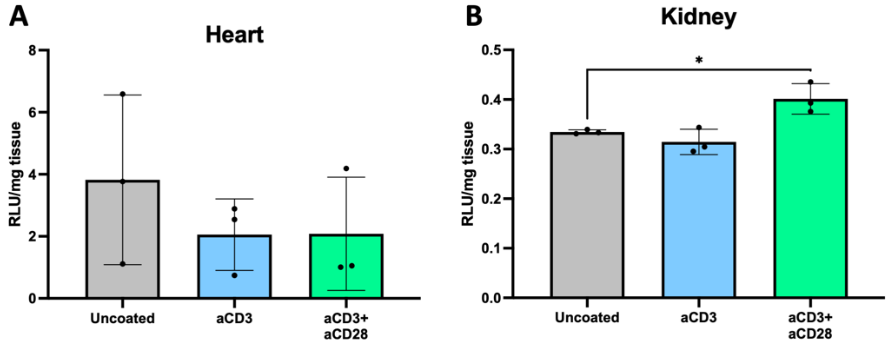

Figure S7. Luciferase signal in heart and kidney. (A) Luminescence signal was analyzed in the heart 24 hours after 10 μg tPNPs delivering luciferase-encoding mRNA were administered intravenously to albino C57BL/6 mice. (B) Luminescence signal was analyzed in the kidney, with aCD3+aCD28 tPNPs demonstrating higher transfection compared to uncoated and aCD3 NPs. Whole organ transfection was analyzed using a one-way ANOVA with Tukey's multiple comparisons test, $^{*}P < 0.05$.

	Group	No Treatment	Uncoated	aCD3	aCD3+aCD28
Spleen Total RLU	Mouse 1	310.4	26146.66667	199500	65046.66667
	Mouse 2	370.6	14296.66667	322033.3333	93886.66667
	Mouse 3	329.7	15976.66667	127766.6667	106233.3333
	Average	336.9	18806.66667	216433.3333	88388.88889
Liver Total RLU	Mouse 1	129.3666667	3684	1771	2115.666667
	Mouse 2	127.7	4819	1373.333333	1175
	Mouse 3	122.4	2412.333333	1016.6	1026.4
	Average	126.4888889	3638.444444	1386.977778	1439.022222
Lung Total RLU	Mouse 1	130.3666667	3028.333333	1708.333333	2062.666667
	Mouse 2	134.6	4523.333333	1535.666667	1178.333333
	Mouse 3	115.5	2794.333333	1115	1102.7
	Average	126.8222222	3448.666667	1453	1447.9
Inguinal LN Total RLU	Mouse 1	215.0	185.4	190.3333333	236.4666667
	Mouse 2	159.6	171.4666667	227.2333333	204.7333333
	Mouse 3	181.7	143.5333333	190.3666667	263.3666667
	Average	185.4333333	166.8	202.6444444	234.8555556
Axillary LN Total RLU	Mouse 1	233.7	234.9	203.1333333	151.7666667
	Mouse 2	126.7	253.6	239.3333333	148.1
	Mouse 3	235.8	461.1666667	206.4666667	170.9666667
	Average	198.7333333	316.5555556	216.3111111	156.9444444
Cervical LN Total RLU	Mouse 1	117.8	152.4333333	709.0666667	528.0666667
	Mouse 2	113.6	153.8333333	155.9333333	208.1666667
	Mouse 3	103.4	202.7	149.7	218.9333333
	Average	111.6	169.6555556	338.2333333	318.3888889
Heart Total RLU	Mouse 1	173	238.3	625.4666667	628.2666667
	Mouse 2	108.8	1324	505	183.0666667
	Mouse 3	136.1	629.0666667	156.4666667	154
	Average	139.3	730.4555556	428.9777778	321.7777778
Kidney Total RLU	Mouse 1	155.2	171.0666667	145	173.3
	Mouse 2	170.8	179.9333333	161.4666667	169.3333333
	Mouse 3	137.2	146.7	165.6666667	167.5666667
	Average	154.4	165.9	157.3777778	170.0666667

Figure S8. Total luciferase signal for each organ harvested from tPNP-treated mice at 24 hours (shown in RLU).

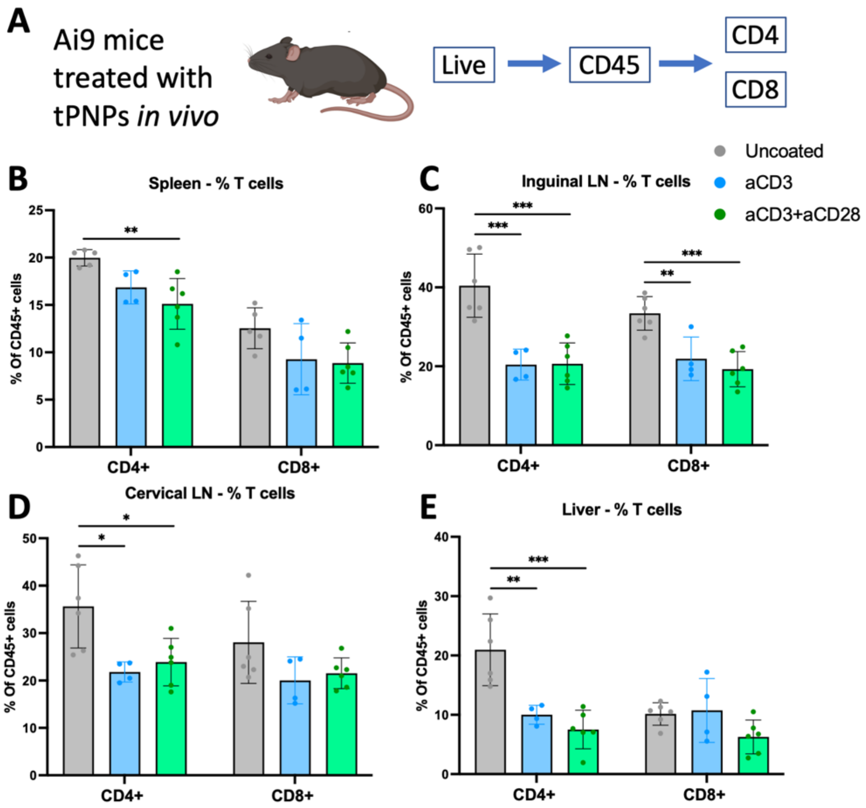

Figure S9: Treatment with tPNPs results in CD4 and CD8 depletion *in vivo.* (A) 10 µg of tPNPs delivering Cre-encoding mRNA were administered intravenously to Ai9 mice. After 24 hours, mice were euthanized and spleens, inguinal LNs, cervical LNs, and livers were isolated, processed. The percent of CD4+ and CD8+ T cells in each organ was analyzed as a subset of CD45+ (all immune) (B-F) Treatment with aCD3 and aCD3+aCD28 tPNPs demonstrates reduced CD4+ and CD8+ T cells in the spleen, inguinal LN, cervical LN, and liver, respectively. Individual statistical analysis for depletion of CD4+ or CD8+ cells in each organ was performed using a one-way ANOVA with Tukey's multiple comparisons test, $^{*}P < 0.05$, $^{**}P < 0.01$, $^{***}P < 0.001$, and $^{****}P < 0.0001$.

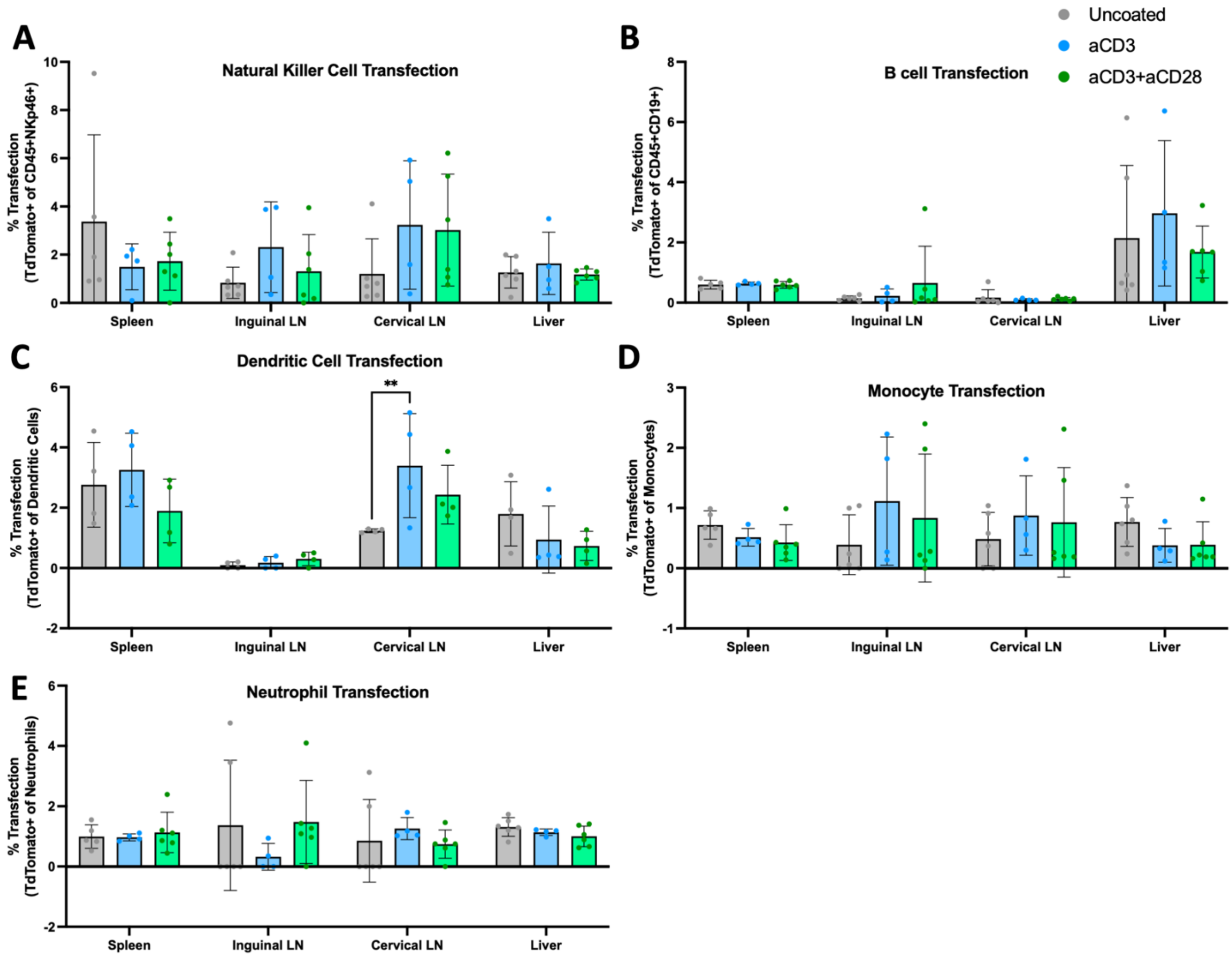

Figure S10. *In vivo* transfection of dendritic cells, B cells, natural killer cells, monocytes and neutrophils in Ai9 mice. 10 μg of tPNPs delivering Cre-encoding mRNA were administered intravenously to Ai9 mice. After 24 hours, mice were euthanized and spleens, inguinal LNs, cervical LNs, and livers were isolated, processed, and analyzed for transfection TdTomato signal across natural killer cells, B cells, monocytes, dendritic cells, and neutrophils (A-E). In all figures, statistical analysis of transfection of the respective cell type in each individual organ was performed using a one-way ANOVA with Tukey's multiple comparisons test, $^{*}P < 0.05$, $^{**}P < 0.01$, $^{***}P < 0.001$, and $^{****}P < 0.0001$.

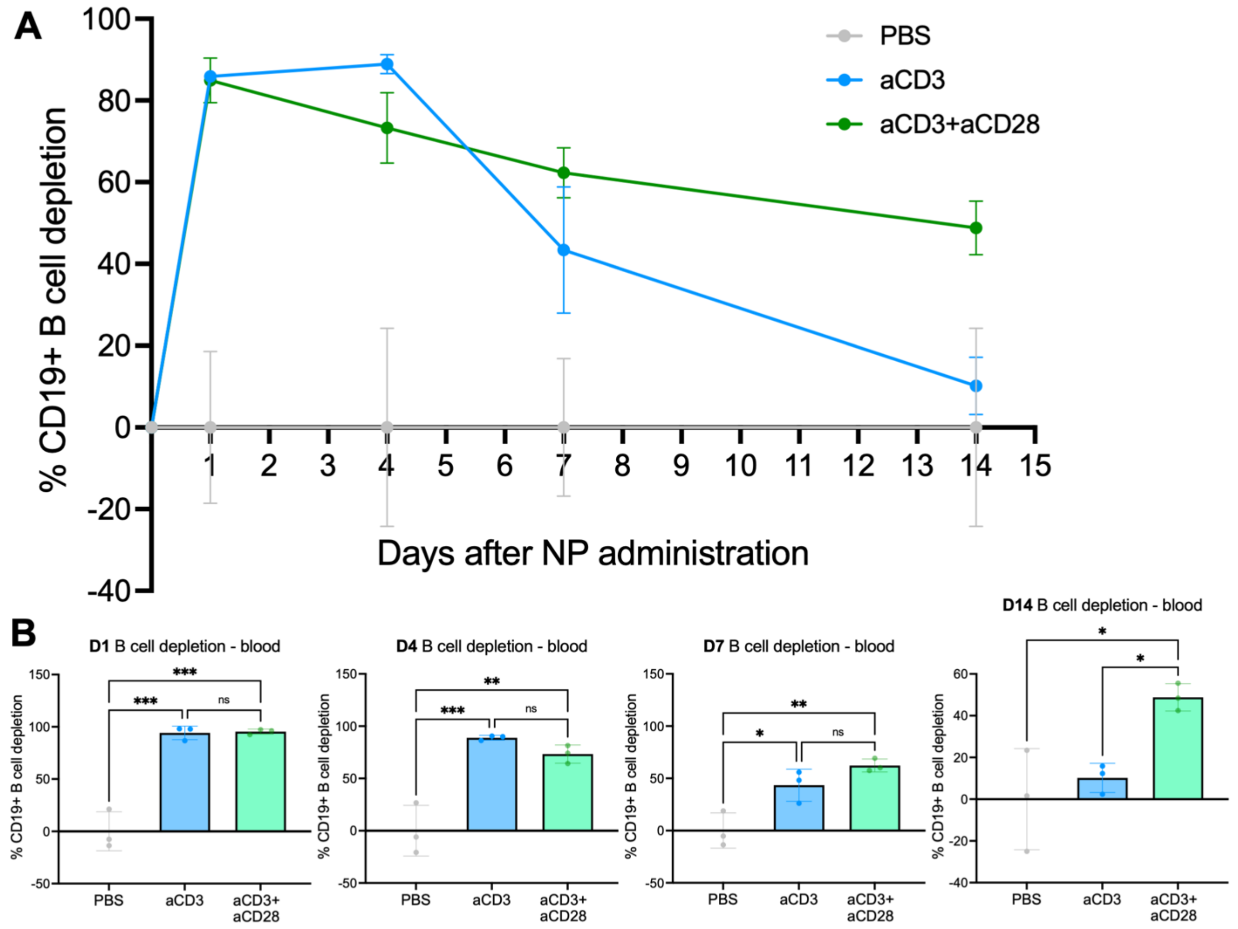

Figure S11. Comparison of *in vivo* B cell depletion kinetics via treatment with aCD3 or aCD3+aCD28 CD19 CAR mRNA tPNPs. (A) aCD3 or aCD3+aCD28 CD19 CAR mRNA-tPNPs were injected to B6 mice on day 0. Mice were then bled via cheek bleed on day 0 (pre-injection), 1, 4, 7, or 14. Bloods were processed and stained for viability, CD45, and CD19 to assess B cell depletion over time. (B) Level of B cell depletion was analyzed between no treatment (PBS), aCD3, and aCD3+aCD28 CAR tPNPs at each specified timepoint. Statistical analysis was performed using a one-way ANOVA with Tukey's multiple comparisons test, $^{*}P < 0.05$, $^{**}P < 0.01$, and $^{***}P < 0.001$.

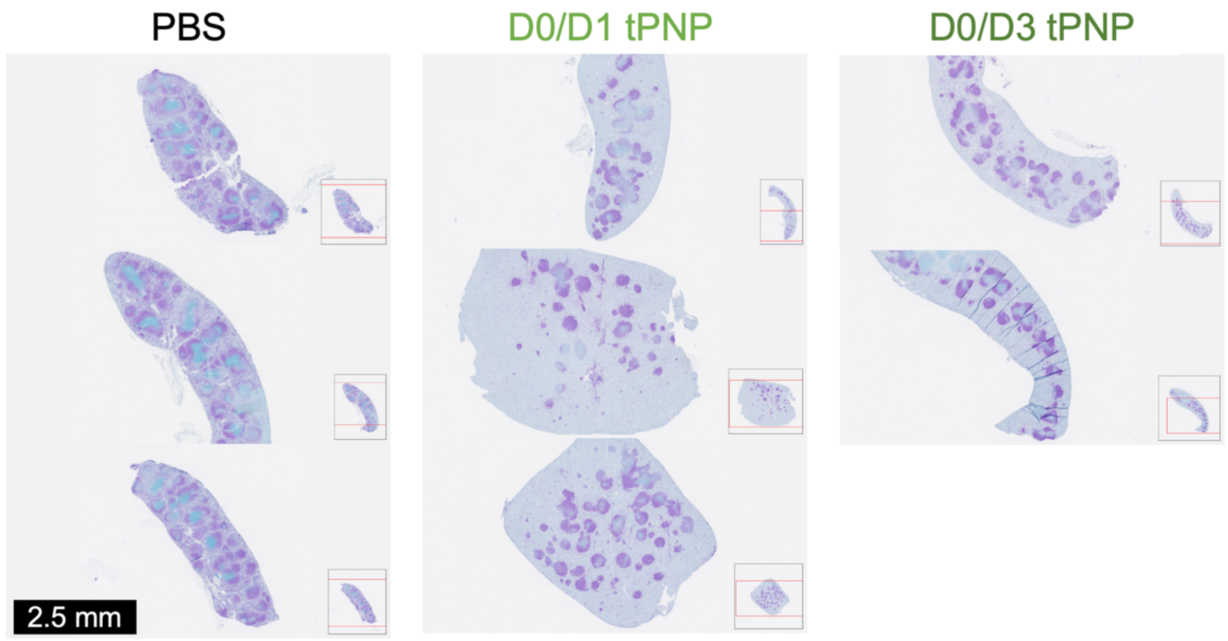

Figure S12: Immunohistochemistry stains of all spleens from mice treated with multiple tPNP doses. B cells are stained in purple and T cells are stained in blue. All IHC stains are shown at 1.25x magnification. Scale bar is representative of 2.5 mm.

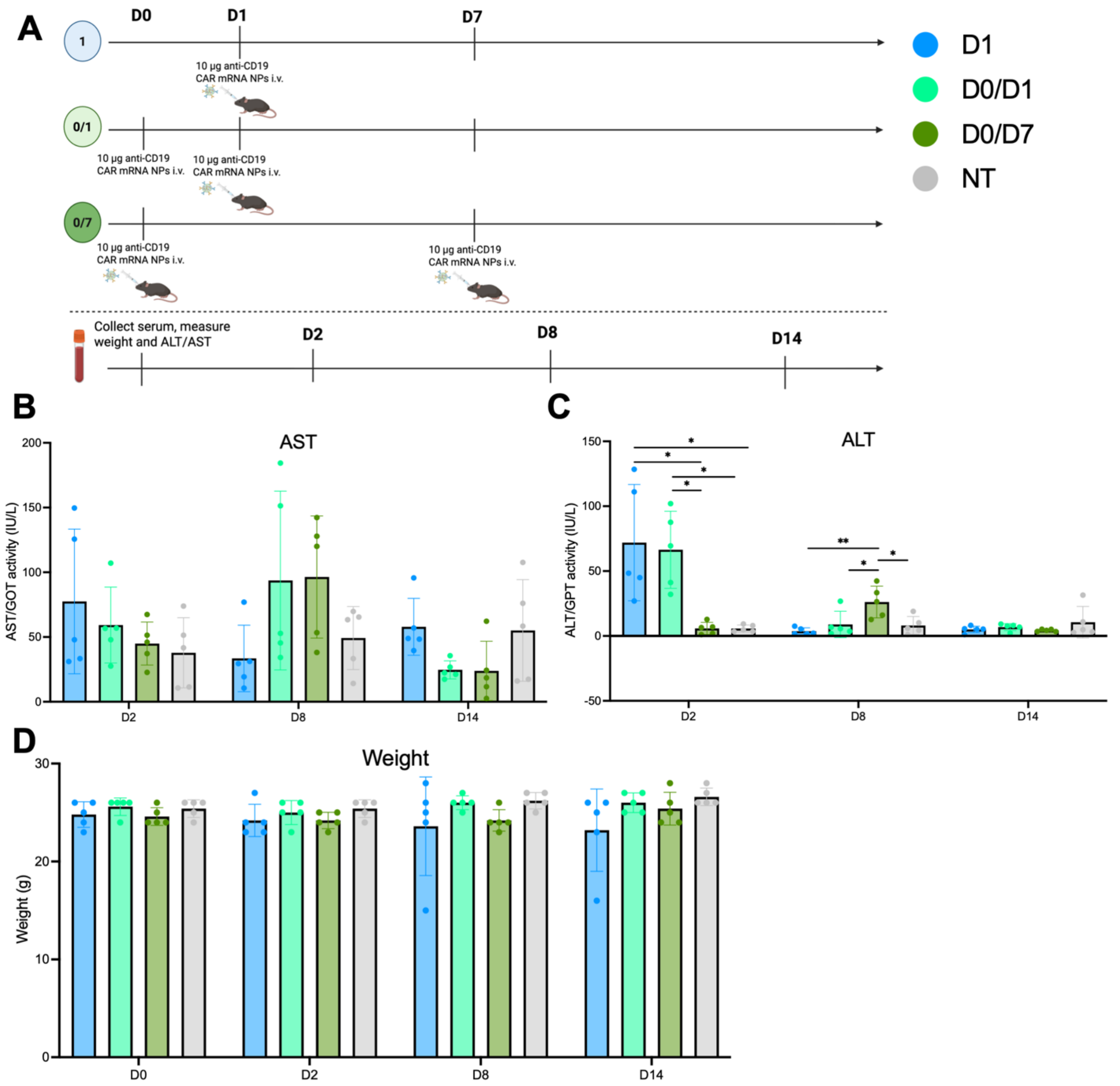

Figure S13. Evaluation of tPNP tolerability across repeated doses. (A) Mice were treated with tPNPs on either D1 only, D0 and D1, or D0 and D7. On D2, D8, and D14, mice serum plasma was isolated for measurement of AST and ALT levels, and mouse weight was measured. All metrics were compared to an age-matched no-treatment (NT) group. (B) Measurement of AST activity at the indicated timepoint across all treatment groups. (C) Measurement of ALT activity at the indicated timepoint across all treatment groups. (D) Measurement of mouse weight at D0, D2, D8, and D14. Statistical analysis was performed using a one-way ANOVA for each individual timepoint with Tukey's multiple comparisons test, $^{*}P < 0.05$ and $^{**}P < 0.01$.

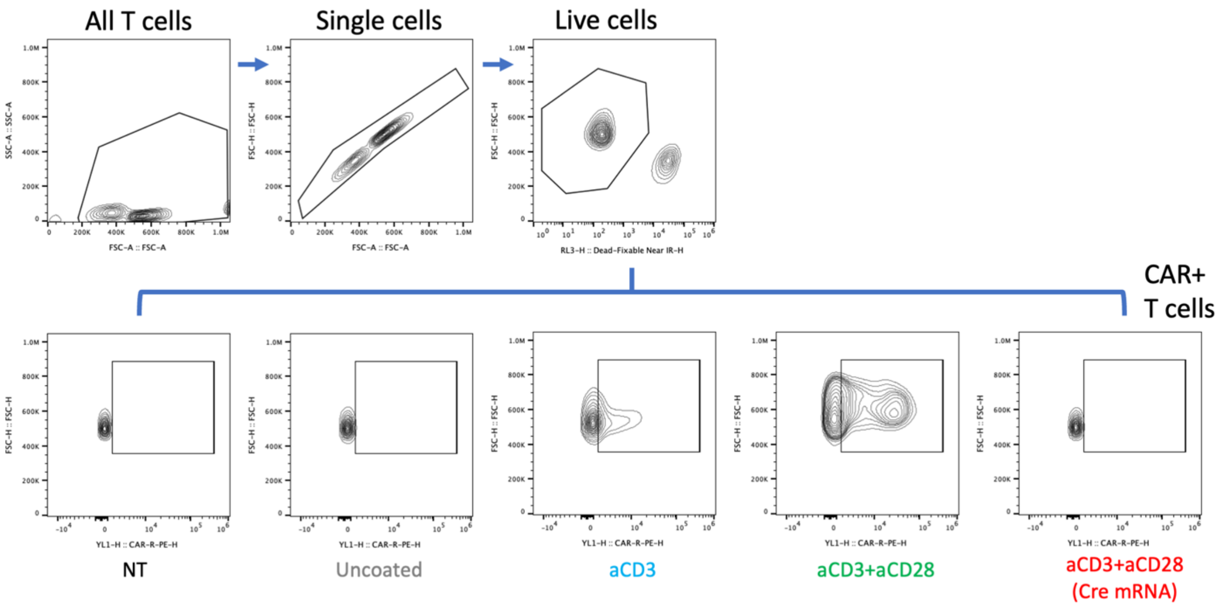

Figure S14: Gating strategy for *in vitro* CAR expression. CAR expression after *in vitro* transfection with CAR mRNA tPNPs was gated relative to a non-treated control group. CAR expression was measured as a subset of live cells, with standard doublet exclusion.

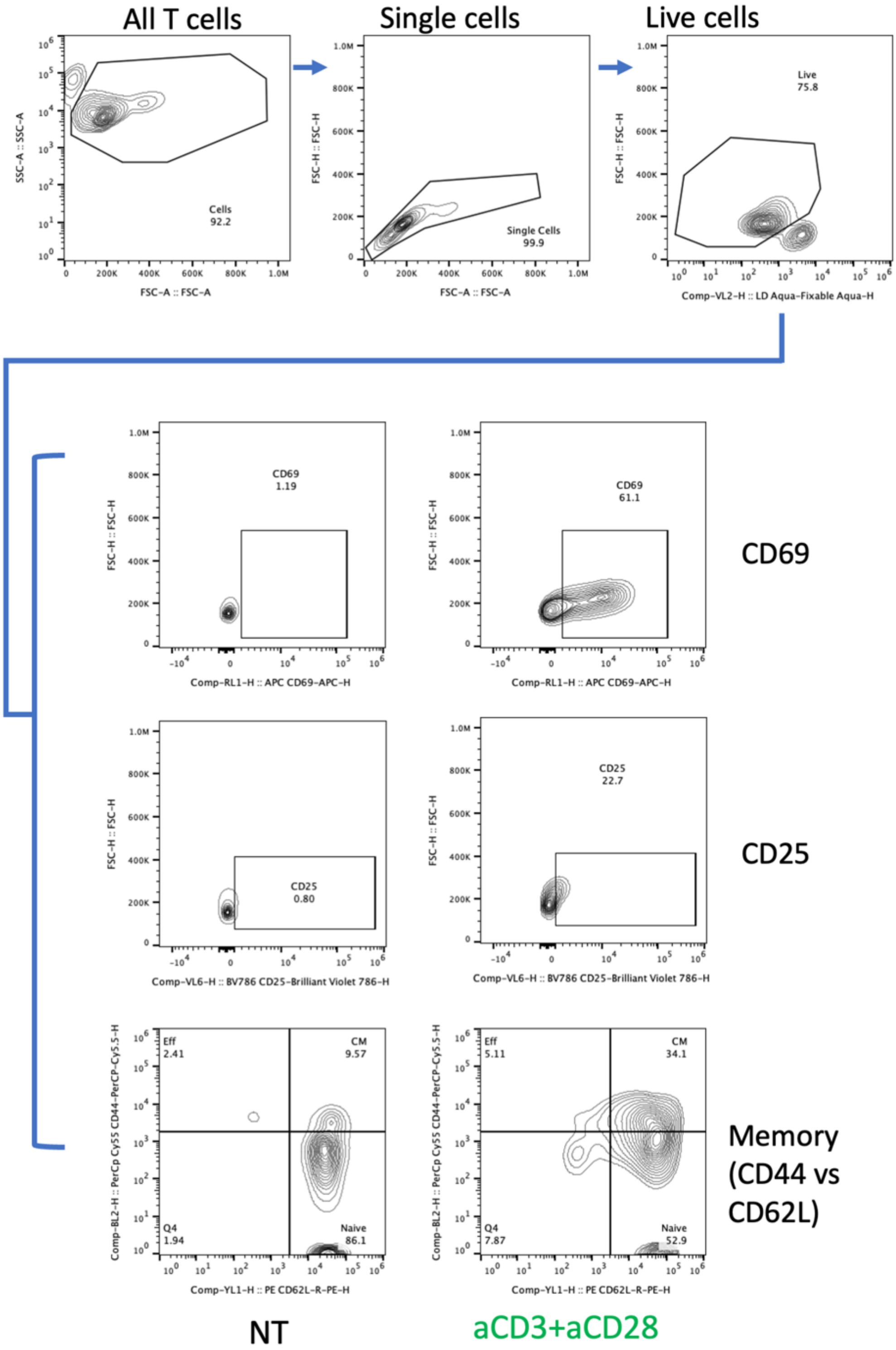

Figure S15: Gating strategy for *in vitro* T cell phenotype. CD69, CD25, and CD44/CD62L expression after *in vitro* transfection with CAR mRNA tPNPs was gated relative to a non-treated control group. Phenotypic markers were measured as a subset of live cells, with standard doublet exclusion.

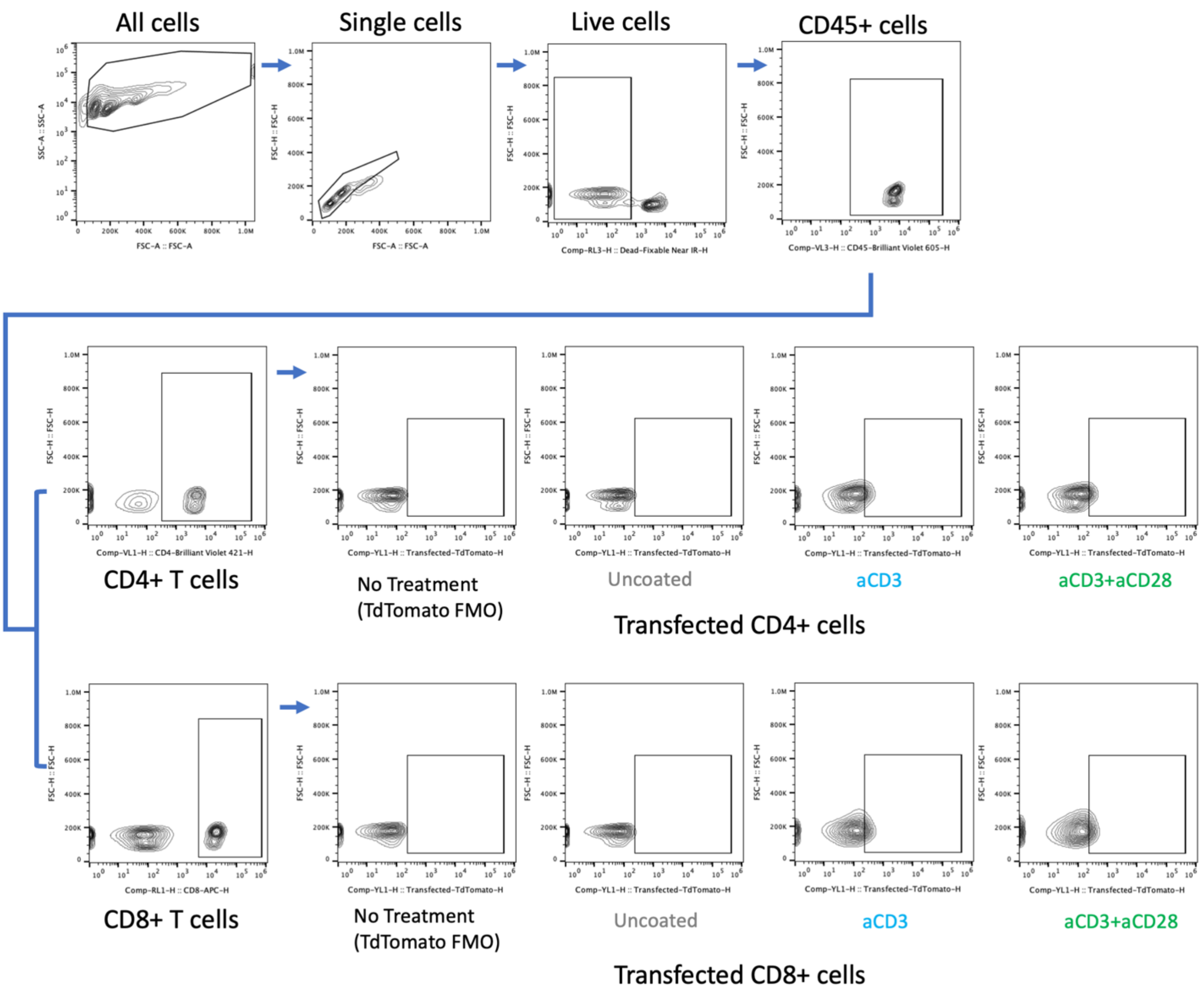

Figure S16: Gating strategy for *in vivo* TdTomato expression in Ai9 mice after Cre mRNA NP administration for T cell panels. TdTomato expression after *in vivo* T cell transfection with Cre mRNA tPNPs was gated relative to a non-treated control group. TdTomato expression was measured as a subset of either CD4+ or CD8+ T cells, which were gated as a subset of CD45+ immune cells and live cells, with standard doublet exclusion.

Gating phenotype on transfected (TdTomato+) CD4 or CD8 cells – **see figure S12**

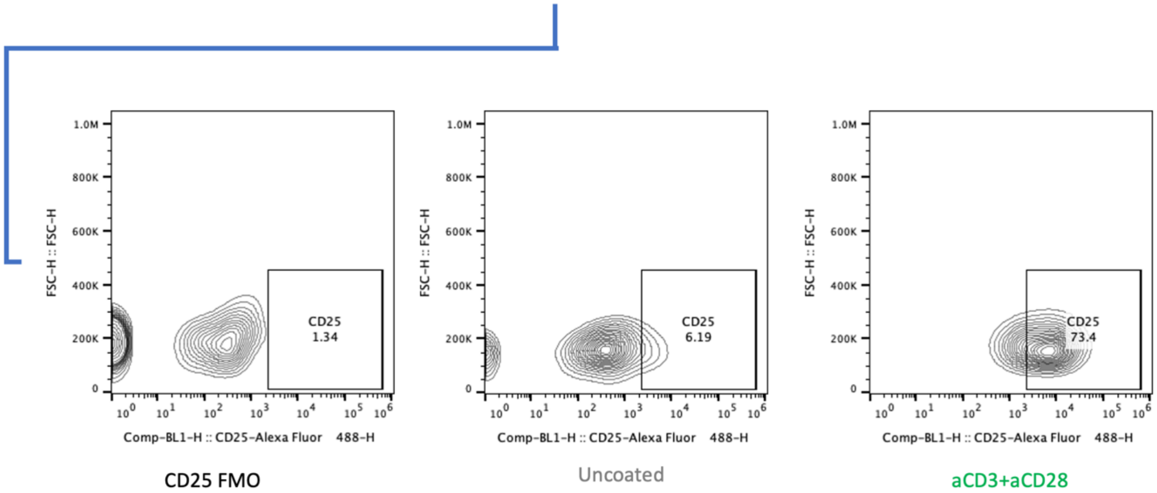

Figure S17: Gating strategy for *in vivo* CD25 expression of TdTomato+ T cells. TdTomato+ CD4+ and CD8+ cells were identified using gating outlined in Figure S12. After identifying TdTomato+ T cells, CD25 expression was measured relative to a CD25 fluorescence minus one (FMO) sample.

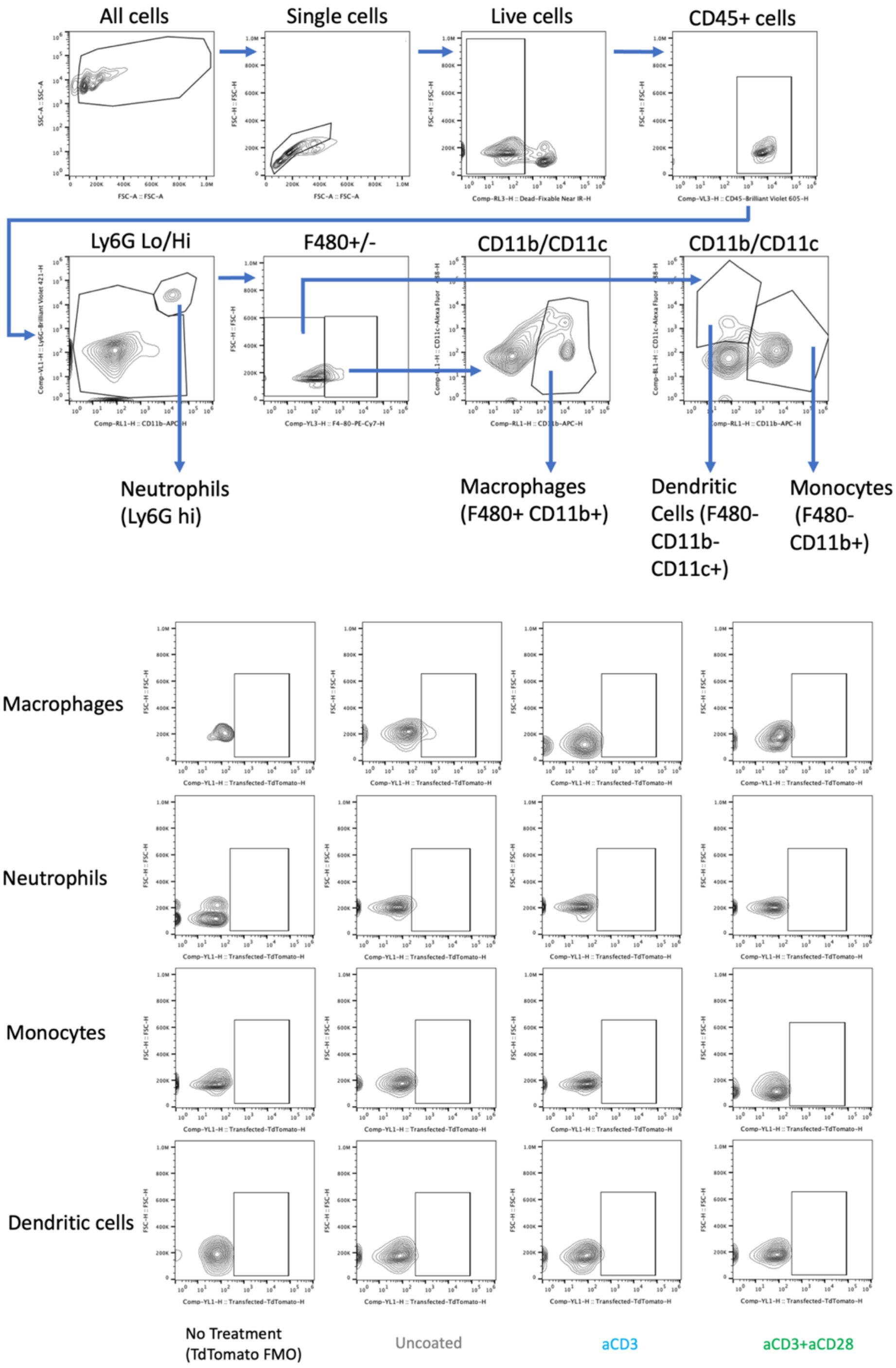

Figure S18: Gating strategy for *in vivo* TdTomato expression in Ai9 mice after Cre mRNA NP administration for myeloid cell panels. TdTomato expression after *in vivo* myeloid cell transfection with Cre mRNA tPNPs was gated relative to a non-treated control group. TdTomato expression was measured as a subset of macrophages, neutrophils, monocytes, or dendritic cells. Myeloid subsets were characterized using the following gates: macrophages (Ly6G lo, F480+CD11b+), neutrophils (Ly6G hi), monocytes (Ly6G lo, F480-CD11b+), dendritic cells (Ly6G lo, F480-CD11b-CD11c+). All myeloid cell markers were gated as a subset of CD45+ immune cells and live cells, with standard doublet exclusion.

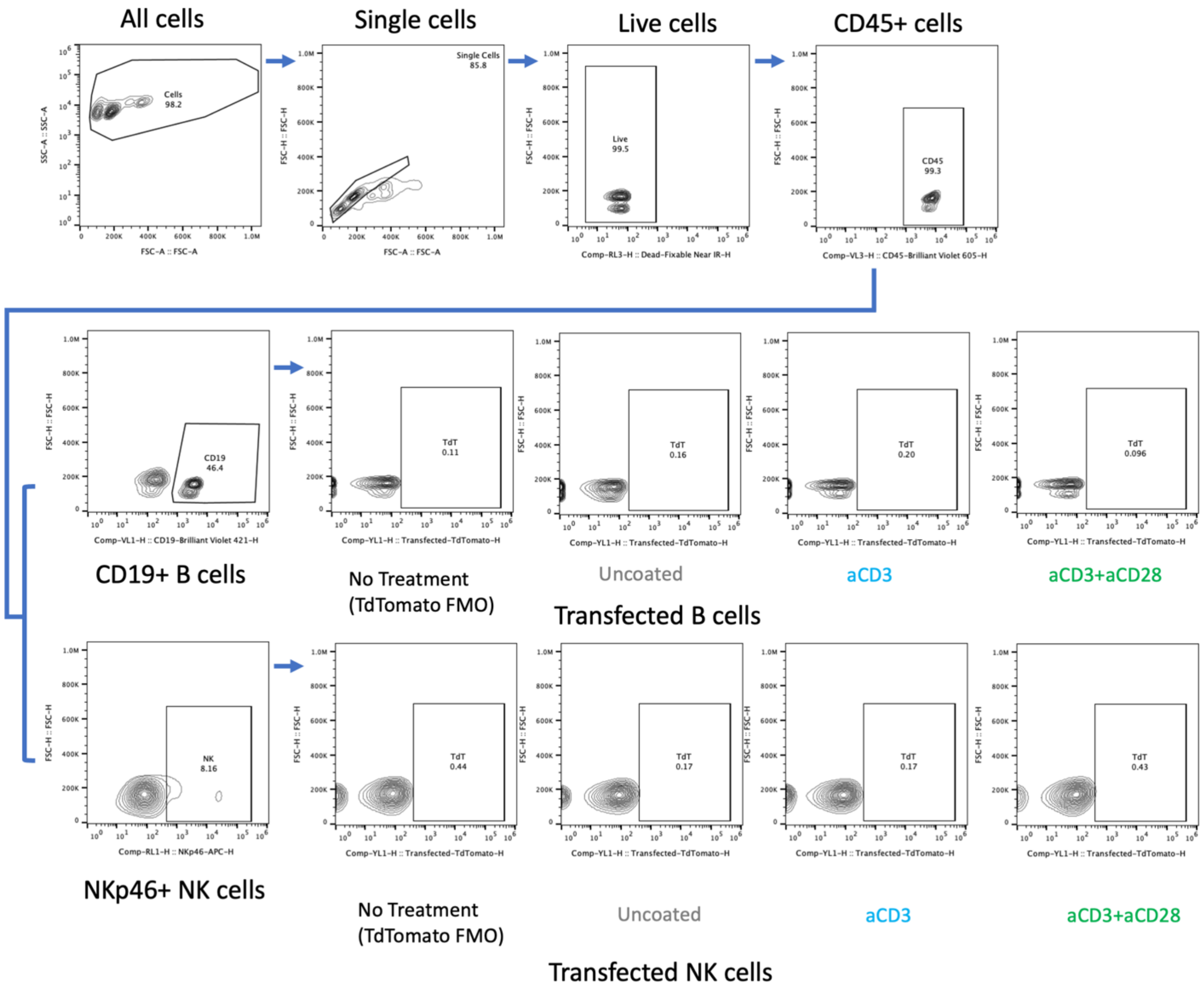

Figure S19: Gating strategy for *in vivo* TdTomato expression in Ai9 mice after Cre mRNA NP administration for lymphoid cell panels TdTomato expression after *in vivo* lymphoid cell transfection with Cre mRNA tPNPs was gated relative to a non-treated control group. TdTomato expression was measured as a subset of natural killer cells (NKp46+) and B cells (CD19+). All lymphoid cell markers were gated as a subset of CD45+ immune cells and live cells, with standard doublet exclusion.

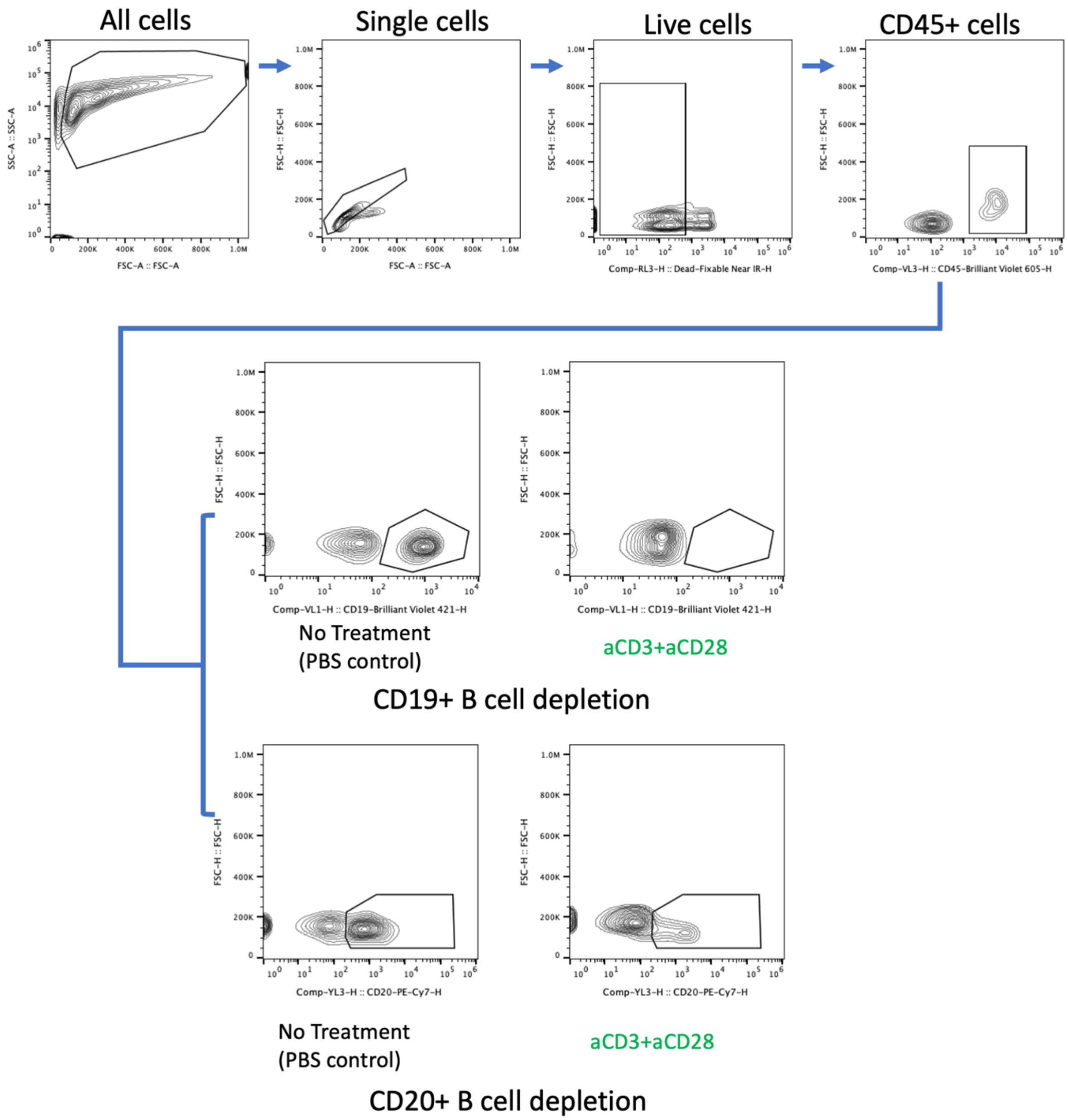

Figure S20: Gating strategy for *in vivo* B cell depletion after CD19 CAR mRNA NP administration. B cell depletion was measured relative to a non-treated control group, whereby the percent of B cells in the control group was measured as the baseline level of B cells. The percent B cell depletion in the blood or spleen sample from a PBS- or CAR mRNA tPNP-treated mouse was then calculated using the following formula:

$$\frac{(Baseline\ \%\ of\ B\ cells - \ \%\ of\ B\ cells\ in\ sample)}{Baseline\ \%\ of\ B\ cells} * 100$$

B cell depletion was measured using both CD19 and CD20 as B cell markers, which were gated as a subset of CD45+ immune cells and live cells, with standard doublet exclusion.
